# The effect of Nrf_2_ deletion on the proteomic signature in a human colorectal cancer cell line

**DOI:** 10.1186/s12885-022-10055-y

**Published:** 2022-09-13

**Authors:** Omid Cheraghi, Bahareh Dabirmanesh, Farideh Ghazi, Massoud Amanlou, Mona Atabakhshi-kashi, Yaghoub Fathollahi, Khosro Khajeh

**Affiliations:** 1 Department of Biochemistry, Faculty of Biological Science, Tarbiat Modares University, P.O. Box: 14115-175, Tehran, Iran; 2 Department of Medical Genetics and Molecular Biology, School of Medicine, Iran University of Medical Sciences, Tehran, Iran; 3 Department of Medical Chemistry, Faculty of Pharmacy and Pharmaceutical Science, Tehran, Iran; 4 CAS Key Laboratory for Biomedical Effects of Nanomaterials & Nanosafety, CAS Center for Excellence in Nanoscience, National Center for Nanoscience and Technology, Beijing, 100190 China; 5 Department of Medical Physiology, Faculty of Medical Science, Tarbiat Modares University, Tehran, Iran

**Keywords:** Nrf_2_, MAPK pathway, Mitochondria, Proteomics

## Abstract

**Background:**

Colorectal cancer is one of the most common cancer and the third leading cause of death worldwide. Increased generation of reactive oxygen species (ROS) is observed in many types of cancer cells. Several studies have reported that an increase in ROS production could affect the expression of proteins involved in ROS-scavenging, detoxification and drug resistance. Nuclear factor erythroid 2 related factor 2 (Nrf_2_) is a known transcription factor for cellular response to oxidative stress. Several researches exhibited that Nrf_2_ could exert multiple functions and expected to be a promising therapeutic target in many cancers. Here, Nrf_2_ was knocked down in colorectal cancer cell line HT29 and changes that occurred in signaling pathways and survival mechanisms were evaluated.

**Methods:**

The influence of chemotherapy drugs (doxorubicin and cisplatin), metastasis and cell viability were investigated. To explore the association between specific pathways and viability in HT29-Nrf_2_^−^, proteomic analysis, realtime PCR and western blotting were performed.

**Results:**

In the absence of Nrf_2_ (Nrf_2_^−^), ROS scavenging and detoxification potential were dramatically faded and the HT29-Nrf_2_^−^ cells became more susceptible to drugs. However, a severe decrease in viability was not observed. Bioinformatic analysis of proteomic data revealed that in Nrf_2_^−^ cells, proteins involved in detoxification processes, respiratory electron transport chain and mitochondrial-related compartment were down regulated. Furthermore, proteins related to MAPKs, JNK and FOXO pathways were up regulated that possibly helped to overcome the detrimental effect of excessive ROS production.

**Conclusions:**

Our results revealed MAPKs, JNK and FOXO pathways connections in reducing the deleterious effect of Nrf_2_ deficiency, which can be considered in cancer therapy.

**Supplementary Information:**

The online version contains supplementary material available at 10.1186/s12885-022-10055-y.

## Introduction

Colorectal cancer is known as one of the most common diagnosed cancers that its survival rate mainly depends on the stage of disease at diagnosis and degree of drug resistance. Elevated production of ROS has been reported in almost all cancer cells due to an increase in growth factor stimulation, mitochondrial dysfunction, higher proliferation and metabolic activity [1].

Low level of ROS plays an important role in tumor signal transfer, proliferation and metastasis but its high concentration is reported to induce cytotoxicity. Hence, the dual role of ROS level in tumor cells could be critical in anticancer therapy. The mechanism of elevating ROS to target cancer cells is via either increasing the generation of ROS through exogenous agents or inhibiting the antioxidant systems [2]. Cells normally counteract the deleterious effects of ROS through the activation of nuclear factor erythroid 2-like 2 (*NFE2L2,* Nrf_2_). Under normal condition, Nrf_2_ is inactive and bound to two molecules of Keap1 which is located in the cytoplasm.

As the oxidative stress increases, Keap1 reactive cysteine residues will be oxidized leading to conformational changes that inhibits binding of Nrf_2_ to KEAP1 and allows Nrf_2_ to escape ubiquitination. Therefore, newly translated Nrf_2_ could bypass KEAP1 and translocate into the nucleus where it transcribes its downstream targets [3].

Nrf_2_ has been shown to control the expression of fundamental components of the glutathione (GSH) and thioredoxin (TXN) antioxidant system. In addition to GSH and SOD1, Nrf_2_ has been exhibited to be crucial in maintaining the redox homeostasis of the cell and regulating the cellular antioxidants. It can also promote the expression of enzymes involved in NADPH regeneration, ROS reduction, xenobiotic detoxification and heme metabolism [4].

Recently, accumulating evidence has shown that metabolic reprogramming is tightly connected to the redox homeostasis. Because cancer cells generally exhibit much higher ROS levels than normal cells [5], thus they are more dependent on antioxidant systems and alteration of specific metabolic pathways. Hence, disrupting Nrf_2_ could be a potential therapeutic strategy against cancer [6, 7].

Recently, many researchers have demonstrated the crucial links between Nrf_2_ cytoprotection and many signaling pathways. To survive increased ROS level, cancer cells activate many signaling pathways such as Mitogen-activated protein kinase (MAPK), FOXO and Notch1 [8]. MAPK cascade (MAP4Ks, MAP3Ks, MAP2Ks and MAPKs) has been reported to be involved in many cellular responses, like proliferation, apoptosis, angiogenesis and cell migration [9].

Based on many evidences from various literatures we were convinced to evaluate and unravel the role of Nrf_2_ in regulating enzymes, signaling pathways, transporters and ROS production in a colorectal cancer cell line, HT29. For this reason, stable Nrf_2_ knock down cells were first generated. Then cellular cytotoxicity and metastatic properties were examined in the presence of two chemotherapy drugs. Due to the capability of omics approaches in providing comprehensive insights on unknown biological mechanisms, here, Nano-flow LC-MS/MS was used to study the proteome of both HT29 and Nrf_2_ knock down HT29. Western blotting, real time PCR, enzyme assay and antioxidant capacity validated the proteomic results. Heatmap, KEGG pathway and Gene ontology of differentially expressed proteins were analyzed and finally protein-protein interaction network was provided to clarify complexity.

## Methods

### Cell culture

Human colorectal cancer cell line, HT29, was obtained from Pasteur Institute (Tehran, Iran). This cell line was cultured in RPMI-1640 medium (Gibco) and supplemented with 10% FBS (Gibco) and 100 U/ml of penicillin-streptomycin (Sigma). Cells were grown in standard condition (5% CO_2_, 37 °C and humidified atmosphere). The medium was changed every 3 days and passaged using 0.25% Trypsin–0.01% EDTA. For all experiments, under sixth passage cells were used.

### Generation of *NFE2L2-*knocked down cell line

In order to knock down Nrf_2_, we used Santa Cruz Nrf_2_ shRNA plasmid (sc-37,049-SH) and used the manufacturers’ protocol. Briefly, 1.2 × 10^5^ cells were seeded in 12 well plate and waited until cells reached to 70% confluence. Subsequently, 1 μg of plasmid and 4 μl TurboFect Transfection Reagent (Thermo Scientific; R0533) were diluted in 100 μl serum-free DMEM. After 15 minutes, the DNA/reagent complex was added to the wells drop wise and incubated in standard condition for next 48 h. Then, the cells were exposed to puromycin selection media for 14 days. To confirm the gene knock down, real time PCR was used (HT29- Nrf_2_^−^). In addition, shRNA plasmid alone transfection was used as a mock control (Santa Cruz; sc-108,060) [10].

### RNA extraction and real time PCR

Total RNA was extracted from the cells by spin column kit (Favorgen; FABRK 001) and its quality was examined (A260/280 ratio ≥ 1.9). One microgram of total RNA was used for cDNA synthesis (by PrimeScript; 6110B). qPCR reaction was carried out by using SYBR Green PCR Master Mix in Step one Real Time PCR instrument (Applied Biosystems) in 95 °C/5 min for initial denaturation then 95 °C/10s, 58 °C/30s and 72 °C/30s for 40 cycles. Analysis carried out by 2^-ΔΔCt^ method with Beta-2 Microglobulin (β2M) as internal control. Primers were designed by PerlPrimer software as follows;

5´-F, TTCCCGGTCACATCGAGAG-3´, 5´-R, TCCTGTTGCATACCGTCTAAATC-3´ for NFE2L2, 5´-F, GACCCATGACACCAAGGA-3´, 5´-R, GCTGAGTGTAAGGACCCA-3´ for HO-1 and 5´-F, AGGCTATCCAGCGTACTCC-3´, 5´-R, ATGTCGGATGGATGAAACCC-3´ for β2M.

### Viability and metastatic properties

Cellular cytotoxic response against two different chemotherapy drug, doxorubicin and cisplatin, was evaluated by MTT (methylthiazol tetrazolium bromide) assay. 2 × 10^4^ Cells were plated in 96 well cell culture plate containing 200 μl 10% FBS media. After 24 or 48 h, different concentration of drugs were subjected to HT29 and HT29-Nrf_2_^−^cell lines. Twenty microliter of MTT (5 mg/ml) (Sigma) was added to each and incubated for 4 h in standard condition. Finally, the cells supernatant were changed with DMSO and absorbance read at 570 nm (with reference wavelength of 630 nm) in ELISA plate reader (BioTek).

The metastasis properties of cells were investigated using the scratch assay. After reaching desired confluency, a monolayer of cells was scratched to create a cell-free line by a yellow tip, and then cells were treated with 0.6 μM of doxorubicin and 125 μM cisplatin for 24 and 48 hours. Distances of two edges were measured using ImageJ 1.48 software [11].

#### Non enzymatic and enzymatic antioxidant assay

To measure antioxidant and prooxidant balance, cells were lysed by 0.5 ml of ice-cold lysis buffer (Cat No: FNN001; Invitrogen) and the bradford method were done to measure the total protein content. Then commercially available colorimetric kit was used to determine the malondialdehyde (MDA) (ZellBio; MDA48), total antioxidant capacity (TAC) (Randox; NX2332) based on ABTS (Azino ethylbenzthiazoline sulphonate) oxidation, super oxide dismutase (SOD) (ZellBio; SOD48), glutathione peroxidase (GPX) (ZellBio; GPX48), catalase (CAT) (ZellBio; CAT48), total glutathione (GSH) (Sigma; CS0260) and H2O2 (Sigma, MAK165) assay according to the manufacturer’s instruction.

#### Nano-flow LC-MS/MS proteomic procedure

In bottom-up proteomics approaches, Nanoflow liquid chromatography combined with high-resolution mass spectrometry (Nano-flow LC-MS/MS), is 100 times more sensitive than traditional LC-MS. Low column internal diameter (ID; 75 μM) makes it a useful technique for identification of cellular proteome changes [12].

##### Protein extraction and digestion

After lysing cells in 8 M urea/0.1 M Tris-HCl, pH 8.0 with protease inhibitor cocktail (Roche), extracted proteins were reduced in 10 mM DTT (Dithiothreitol) for 2 h and alkylated in 20 mM iodoacetamide for 30 min at 25 °C in dark condition. Then, the protein solution was diluted 1:5 with 50 mM TEAB (triethylammonium bicarbonate) and digested overnight with trypsin (1:50 ratio) at 37 °C. At the end, the digestion was quenched via acidification with formic acid. After that, we used OASIS HLB column to desalt the digestion, and finally peptides were eluted with 60% acetonitrile and lyophilized via vacuum centrifugation.

##### Peptide labeling with TMT

Prior to Tandem Mass Tag (TMT) labeling, the dried peptide was resolved in TEAB (Triethylammonium bicarbonate buffer) and then 100 μg protein of each biological replicates were labeled with TMT-130 and TMT-131 (Thermo Scientific) according to the manufacturer’s protocol.

##### Peptides fractionation by pH reverse phase chromatography

For desalting the mixed TMT-labeled peptides, Sep-Pak Vac C18 SPE cartridges (Waters, Massachusetts, USA) was used following drying in a vacuum concentrator. Next, this peptide suspended in 2% acetonitrile, pH 10 (solution A) and loaded onto YMC-Triart C18 basic RP-LC column (250 × 4.6 mm, 5 um particles). For peptide separation, a binary buffer of solution A and B (98% acetonitrile) operated at 0.7 ml/min on L-3000 HPLC System (Rigol). All fractions were collected at 90s intervals and concatenated into 12 post-fractions and lyophilized until nanoLC-MS/MS analysis.

##### LC-MS/MS analysis

All nanoLC-MS/MS experiments were performed on a Q Exactive (Thermo Scientific) equipped with an Easy n-LC 1000 HPLC system (Thermo Scientific). The labeled peptides were loaded onto a 100 μm id× 2 cm fused silica trap column packed in-house with reversed phase silica (Reprosil-Pur C18 AQ, 5 μm, Dr. Maisch GmbH) and then, separated on a 75 μm id× 20 cm C18 column packed with reversed phase silica (Reprosil-Pur C18 AQ, 3 μm, Dr. Maisch GmbH). The peptides bounded on the column were eluted with a 78 min linear gradient. The solvent A consisted of 0.1% formic acid in water solution and the solvent B consisted of 0.1% formic acid in acetonitrile solution. The segmented gradient was 5–8% B, 8 min; 8–22% B, 50 min; 22–32% B, 12 min; 32-95% B, 1 min; 95% B, 7 min at a flow rate of 310 nl/min.

The MS analysis was performed with Q Exactive mass spectrometer (Thermo Scientific). With the data-dependent acquisition mode, the MS data were acquired at a high resolution 70,000 (m/z 200) across the mass range of 300–1600 m/z. The target value was 3.00E+ 06 with a maximum injection time of 60 ms. The top 20 precursor ions were selected from each MS full scan with isolation width of 2 m/z for fragmentation in the HCD collision cell with normalized collision energy of 32%. Subsequently, MS/MS spectra were acquired at resolution 17,500 at m/z 200. The target value was 5.00E+ 04 with a maximum injection time of 80 ms. The dynamic exclusion time was 40s. For nano-electrospray ion source setting, the spray voltage was 2.0 kV; no sheath gas flow; the heated capillary temperature was 320 °C.

The Proteome Discovery (V; 2.2.0.388) was used for protein identification and Percolator for false discovery rate (FDR) analysis of raw data from Q Exactive. For searching of human protein database, we used Uniprot search engine and set some parameters. Trypsin was selected as the enzyme and two missed cleavages were allowed; the mass tolerance of precursor was set to 10 ppm and the product ions tolerance was 0.02 Da.; TMT 2plex (lysine and N-terminus of peptides) and cysteine carbamidomethylation were chosen as fixed modifications and the methionine oxidation was specified as variable modification. The peptides confidence was set as high for peptides filter and FDR < 1% was set for protein identification. Proteins quantification was performed using the ratio of the intensity of reporter ions from the MS/MS spectra. Unique and razor peptides were taken for protein relative quantification. The co-isolation threshold was specified as 50% and average reporter’s S/N value should be above 10. The normalization to the protein median of each sample was used to correct experimental bias and the normalization mode was selected as total peptide amount.

### Heatmap visualization and functional annotation

Hierarchical clustering with Pearson’s correlation of the differently expressed proteins were analyzed by R software. For gene ontology determination, differently expressed proteins were divided into up and down regulated proteins that were then categorized into three groups MF, CC and BP (Molecular function, Cellular component and Biological process). Along with this procedure KEGG enriched pathways were determined by using human Enrich r database [13, 14].and the important terms ranked based on Fisher’s exact test calculated *p*-value. All sections in gene annotation were grouped based on their gene ontology number and *p*-value < 0.05 [15].

### Protein-protein interaction (PPI) network

String webserver (https://string-db.org/) is the most well-known online database for simple and more rapid technique to assess PPI. 172 differently expressed proteins were uploaded and the minimum required interaction score were set to high confidence (0.7). Experiments, databases and text mining were used as active interaction sources. The TSV file was visualized in Cytoscape software (version 3.7.1). In graph theory, the number of connections to each node was defined as degree. The betweenness value for each node was related to its centrality in clusters.

### Western blot

Total proteins were extracted from HT29 and HT29-Nrf_2_^−^ cells with RIPA cell lysis buffer (Cell Signaling) on ice and quantified using Bradford standard technique. One hundred microgram of extracted proteins loaded onto 10% SDS-PAGE and transferred to PVDF membrane. Then PVDF was blocked with 5% BSA for 2 h and then incubated with primary antibodies ERK 1/2 (Santa Cruz, USA, #sc-292,838), Heme Oxygenase 1 (HO-1), (Santa Cruz, USA, #sc-136,960), Ki-67 (Santa Cruz, USA, #sc-23,900), PKLR (Santa Cruz, USA, #sc-166,228), SLC25A27 (UCP4) (Mybiosource, USA, #MBS668834), FOXO3a (Cell Signaling, USA, #2497), cytochrome c (Cyt c) (Santa Cruz, USA, #sc-13,156) and β-actin (Santa Cruz, USA, #sc-130,301) overnight at 4 °C. After several washing with TBST, the PVDF was incubated with secondary mouse IgG (Santa Cruz, USA, #516102) or mouse anti-rabbit (Santa Cruz, USA, #sc-2357) for 2 h at 37 °C. The protein bands were visualized using ECL Detection reagent (Pierce, Rockford, IL, USA). Densitometry band quantification was performed using ImageJ software.

### Statistical analysis

All experiments were performed in triplicate and data were expressed as mean ± SD. Student’s t-test was used to assess the significance of differences in experiments with only two groups and one-way Analysis of variance (ANOVA) was used in experiments with more than two groups. We performed data analysis with GraphPad prism software version 2.02. Statistical differences between groups are indicated as follows: *P < 0.05, **P < 0.01, and ***P < 0.001.

## Results

### Absence of Nrf_2_ on anti-oxidative capacity

**Fig. 1:**
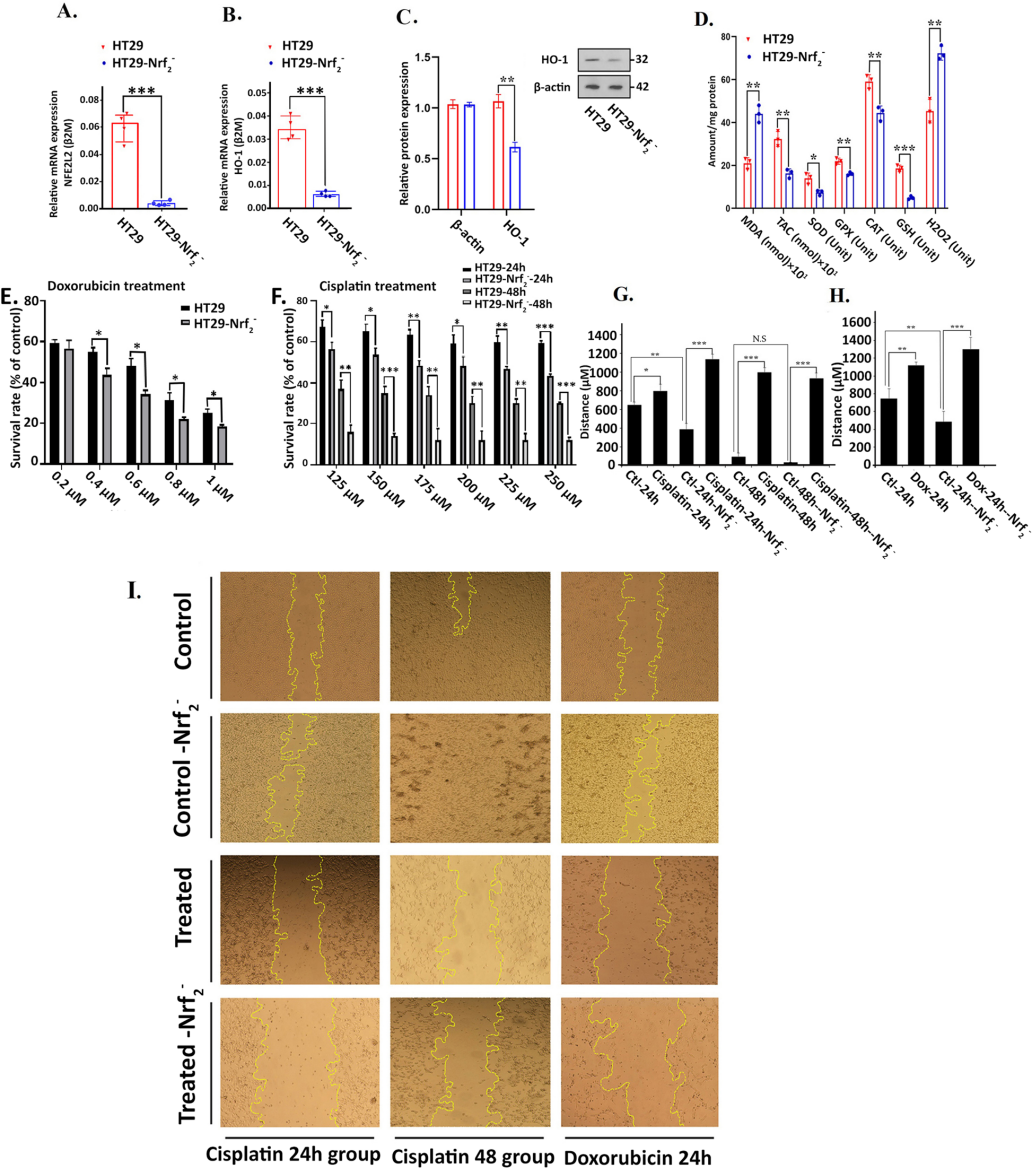
Investigating the fold change expression of (**A**) Nrf_2_ and (**B**) HMOX-I in HT29 and HT29-Nrf_2_^−^ cell line. **C** Western blot data of Nrf_2_ downstream HO-1 gene (**D**) Nrf_2_ related enzyme assay showed a reduction in antioxidant enzymes activity accompanied by an increase in prooxidant markers such as MDA and H2O2. Cytotoxicity effects of (**E**) Doxorubicin and (**F**) Cisplatin treatment on HT29 and HT29-Nrf_2_^−^ cell line, exhibiting a dose- and time-dependent manner. **G, H, I** In-vitro scratch assay to investigate the effect of Doxorubicin and Cisplatin chemotherapy drugs on migration of HT29 cells after 24 and 48 hours treatment. The cells were treated with 125 μM concentration of Cisplatin (for 24 and 48 hours) and 0.6 μM concentration of Doxorubicin drugs (for 24 hours). The HT29-Nrf_2_^−^ cells were more susceptible to drug treatment. Results were expressed as the mean ± SD (*p < 0.05, **p < 0.01, and ***p < 0.001)

Nrf_2_ has been commonly viewed to maintain the oxidant integrity of the cells by regulating the expression of many antioxidant genes. To characterize the colorectal cancer changes associated with Nrf_2_ expression, stable knock down of Nrf_2_ by specific shRNA in HT29 cells was carried out. To verify successful gene knock down, qPCR was used and the relative mRNA level of Nrf_2_ (approximately 17-fold reduction; *p*-value≤0.001) and its downstream gene, heme oxygenase-1 (HO-1), were significantly decreased compared to the control (Fig. 1A–B). Western blot analysis revealed that Nrf_2_ downstream HO-1 expression in HT29-Nrf_2_^−^ cells was significantly less than HT29 cells (Fig. 1C and Fig. 1S in supplementary data).

Nrf_2_ transcription factor modulates the expression of genes involved in stress responses and regulates the cellular defense against toxic and oxidative stress. As shown in Fig. 1D, the activities of seven antioxidant compounds were determined to clarify the effect of Nrf_2_ Knock down on oxidative stress in colorectal cancer cell. Total antioxidant capacity (TAC) (*P* < 0.01), super oxide dismutase (SOD) (*P* < 0.05), glutathione peroxidase (GPX) (*P* < 0.01), catalase (CAT) (*P* < 0.05) and glutathione (GSH) (*P* < 0.01), decreased markedly in HT29-Nrf_2_^−^ cells compared to the control cells. In addition, due to the reduction in antioxidant defense, hydrogen peroxide and malondialdehyde (MDA) (obtained from lipids oxidation) were significantly increased by 1.5 (*P* < 0.05) and 2 (*P* < 0.01) folds, respectively.

### Cell viability and metastatic properties of Nrf2 deficient cells

Owing to the fact that Nrf_2_ has distinctive role in cancer cells viability in the presence of oxidative stimulus, MTT and morphological assays were used to evaluate the HT29-Nrf_2_^−^ cells response to chemotherapy drugs. Therefore, cells were treated with different concentration of doxorubicin (for 24 h) and cisplatin (for 24 and 48 hours) (Fig. 1E and F). As expected, HT29-Nrf_2_^−^ cells were more sensitive to both drugs when compared to HT29. Upon increasing concentrations of DOX, the viability decreased but in the case of Cisplatin, incubation time had a greater effect on toxicity than the concentration. Hence, DOX and cisplatin exhibited dose and time dependent manner, respectively. To investigate the migration capability of HT29 -Nrf_2_^−^ cells, 0.6 μM of doxorubicin (for 24 h) and 125 μM of cisplatin (for 24 h and 48 h) were used for wound healing assay. According to the results, in the absence of drugs, migration of HT29-Nrf_2_^−^ cells towards each other were more than HT29 (*P* < 0.001), however, by the addition of drugs to the culture medium the opposite results were observed and the migration of HT29-Nrf_2_^−^ was reduced when compared to the HT29 (Fig. 1E, F, G, H and I).

### Hierarchical clustering analysis of differently expressed proteins in HT29-Nrf_2_^—^ cells; Heatmap & KEGG pathway

**Fig. 2:**
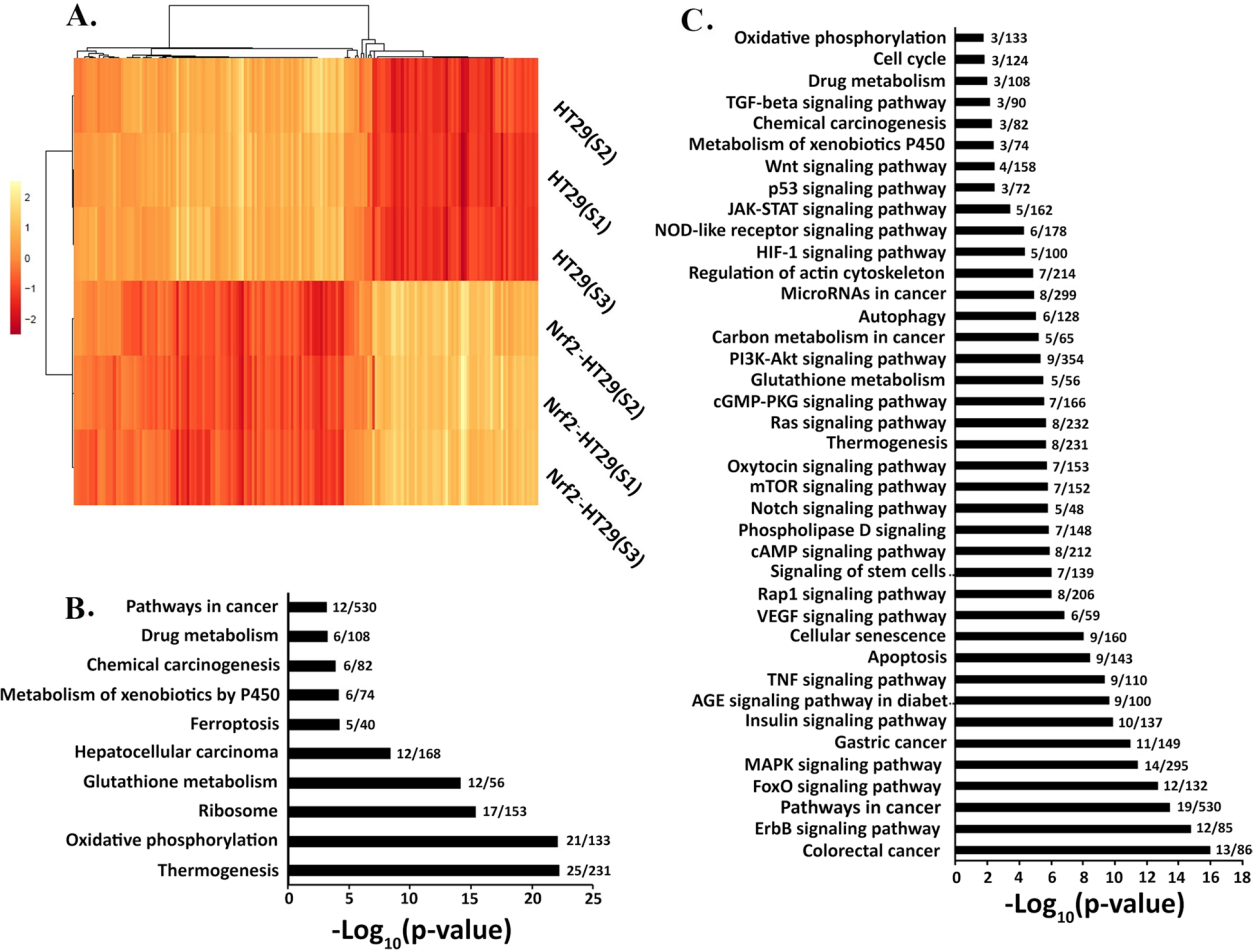
Hierarchical clustering analysis of protein expression. **A** Indicating the differentially expressed proteins in response to the absence of Nrf_2_ in heat map plot. The − 2 is considered as the lowest expression and + 2 presents the highest expression ratio. In HT29-Nrf_2_^−^ cells, two third of proteins were down regulated. **B** Down regulated and (**C**) Up regulated proteins of each cellular pathways were presented as -Log_10_*p*-value in KEGG pathway. The number of overlapped genes in each pathway were added in front of the columns

Nrf_2_ has multifunctional role in cellular antioxidant and surveillance maintenance. Following drug treatment, viability of cells lacking Nrf_2_ decreased about 20% compared to HT29 and it was likely possible that an unknown signaling pathway compensates this deficiency [16]. This phenomenon triggered us to conduct a deep view on some signaling pathways that could help the cells to tolerate against chemotherapy drugs. Therefore, Nano-flow LC-MS/MS proteomic analysis was performed to assess level of oxidative stress and related proteins in the cells. These bottom-up shotgun proteomics identified 5883 unique proteins below 1% FDR. Experiments were conducted in three replicates and robust statistical cutoff value of *p*-value ≤0.05. Among detected proteins, 1280 exhibited less than 0.7 and 977 proteins showed expression greater than 1.5-fold (HT29-Nrf_2_^−^ abundance /HT29 abundance) in their expression. Based on previous literatures, that reported Nrf_2_ as the key component of oxidative stress [17], 171 proteins were identified from differently expressed proteins that were related to oxidative pathways. Within these proteins, 56 proteins were up regulated (ratio ≥ 1.5) and 115 proteins were down regulated (ratio ≤ 0.7). Furthermore, bioinformatic analyses were performed to reveal the altered signaling pathways in Nrf_2_ knocked down cells. Subsequently, hierarchical clustering was done on the proteomic data for HT29-Nrf_2_^−^ and HT29. Figure 2A shows a Heatmap of the correlation matrix across the two groups. As shown in the Fig. 2A, HT29-Nrf_2_^−^ and HT29 showed a clear pattern and samples were clustered closely for each condition.

KEGG analysis (https://amp.pharm.mssm.edu/Enrichr/) [13, 14] was used to analyze the most significantly enriched pathways for the up and down regulated proteins. As shown in Fig. 2B, from 115 down regulated proteins in KEGG enrichment pathway, proteins with the highest *p*-value belonged to thermogenesis, oxidative phosphorylation and ribosome that 25, 21 and 17 proteins overlapped in each term. Among 56 up regulated proteins, molecular signaling pathway especially proteins related to MAPKs and FOXO played an important role within enriched pathways. ErbB, FOXO and MAPKs signaling pathways revealed higher *p*-value versus other signaling pathways (Fig. 2C). A closer look at the overlapping up regulated genes it was noticed that MAPK proteins were distributed remarkably in 30 out of 39 pathways, particularly MAPK3 (in 27 pathways), MAP2K1 (in 25 pathways), Raf1 (in 24 pathways), MAPK9 (in 14 pathways) and BRAF (in 11 pathways) [18].

### Gene Ontology (GO) analysis of differently expressed proteins

**Fig. 3:**
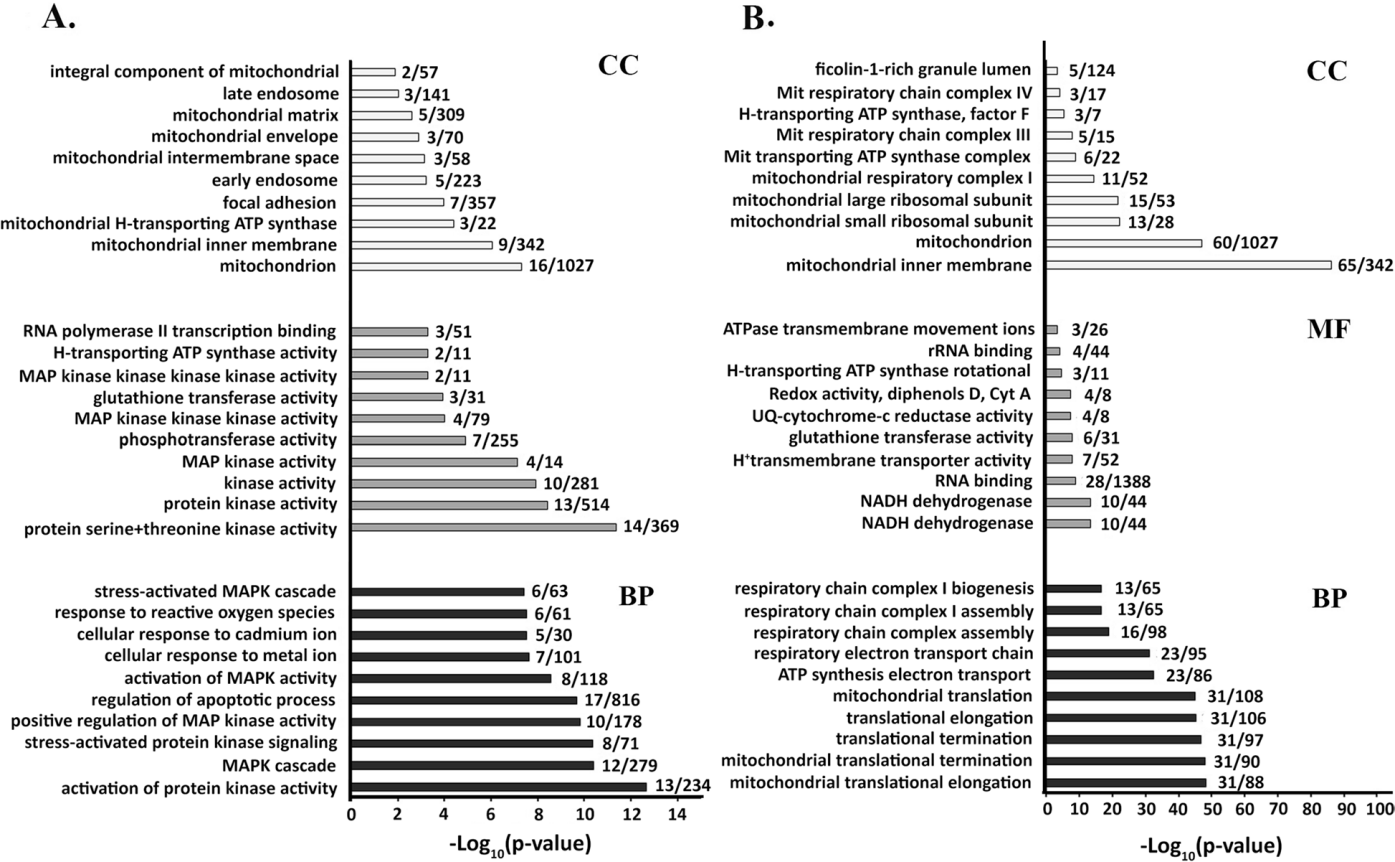
Categorizing gene and function of gene products in three groups; biological process (BP), molecular function (MF) and cellular component (CC) using Gene Ontology (GO) analysis. Top 10 of each process and pathways were analyzed and number of related proteins listed in front of each term in HT29-Nrf_2_^−^ cells. **A** Up regulated and (**B**) down regulated proteins were represented as -Log_10_*p*-value

Enrichment of the differentially expressed proteins was evaluated at the Enrichr website (https://amp.pharm.mssm.edu/Enrichr/) [13]. GO analysis for biological process (BP), molecular function (MF) and cellular component (CC)) were separately performed on 56 up regulated and 115 down regulated proteins. GO enrichment of up regulated proteins showed that 131 terms in BP (14.5%, 131/903), 7 terms in CC category (9.8%, 7/71) and 15 terms in MF (10%, 15/148) have significant value (*p*-value≤0.001). Top ten terms in each category are illustrated in Fig. 3A. In BP (Fig. 3A), the protein kinase activity and MAPKs cascade are the main component that contained 13/234 and 12/279 overlapped proteins in each GO term, respectively. Glutathione transferase activity, ATPase activity and RNA polymerase transcription are three top components of MF category (Fig. 3A) that are related to kinase family similar to BP. In the CC category, the highest number of enriched proteins are localized in mitochondria indicating that MAPK and mitochondria related proteins are mostly affected in Nrf_2_ knock down cells (Fig. 3A).

GO enrichment analysis of down regulated proteins showed that 67 terms from 1099 in BP category, 12 terms from 105 in CC category and 14 terms from 184 in MF category have significant value (*p*-value≤0.001). In BP term (Fig. 3B), the highest *p*-value is related to the translation, ATP synthesis process and respiratory chain complex of mitochondria. In MF the down regulated proteins (Fig. 3B**)** are linked to NADH dehydrogenase activity, RNA binding and five terms are related to respiratory chain function. Therefore, the most altered proteins are associated with mitochondrial function (Fig. 3B).

### Knock down of Nrf_2_ affected antioxidant defense and detoxification in HT29-Nrf_2_

**Fig. 4:**
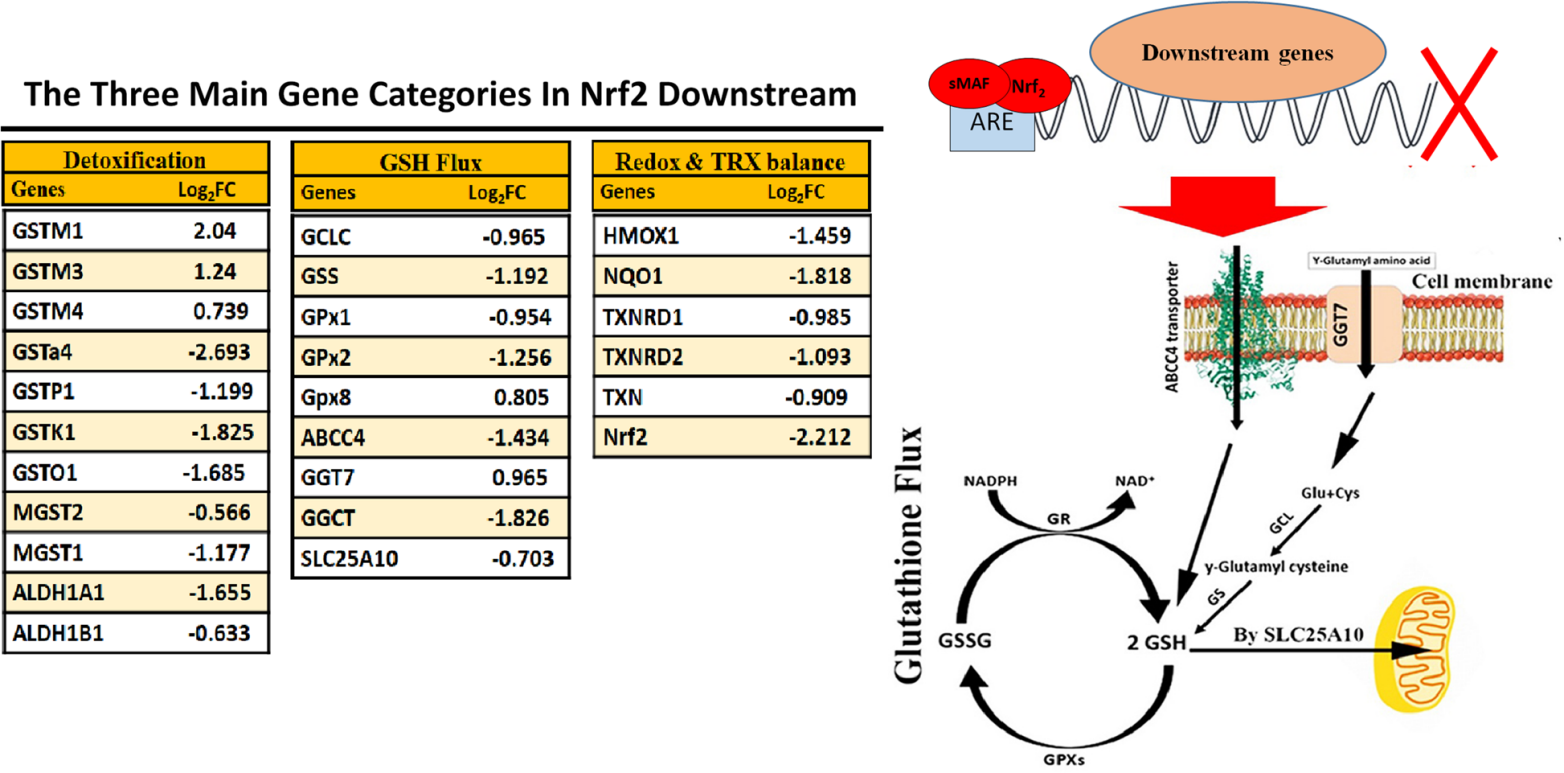
**A** Expression fold change (FC) ratio (HT29-Nrf_2_^−^/HT29) of 26 individual proteins of cell detoxification’s pathways. Almost all proteins were down regulated. **B** The involved proteins in glutathione biosynthesis pathway were presented schematically

Presence of Nrf_2_ affects drug detoxification, glutathione flux and thioredoxin balance (TRX) in cells and organelles, therefore the expression of mentioned pathways was extracted from proteomic data (Fig. 4). Glutathione in cytoplasm can serve as a radical scavenger and detoxifier [19]. Its reduction could lead to an increase in H_2_O_2_ concentration due to unavailability of GSH to convert H_2_O_2_ to H_2_O. This deficiency led to MDA production, a marker of lipid peroxidation. Additionally, GSH could covalently bind to the hydrophobic drugs and prevent them to reach the DNA. It enhances hydrophobic drugs solubility and allows them to be transported across the cell membrane. In addition to antioxidant genes (HMOX1, NQO1, TXNRDs and thioredoxin), the expression of glutathione s-transferases (GSTs) family proteins were also altered in the absence of Nrf_2_. In contrast to GSTMs (GSTM1/3/4) family, expression of cytosolic GSTs (GSTP1, GSTK1, GSTO1, GSTa4) and microsomal GST family (MGSTs) were exclusively decreased as illustrated in Fig. 4A.

Further investigation of the results revealed the reduction of enzymes involved in GSH biosynthesis (Fig. 4B). Glutathione function in cancer development and oxidative stress has been demonstrated previously [20]. Glutathione synthesis is catalyzed by glutathione synthetase (GS) and glutamate-cysteine ligase (GCL) via an ARE-Nrf_2_-dependent pathway. Glutamate cysteine ligase (GCL) is the rate-limiting enzyme of GSH synthesis and it is composed of a catalytic (GCLC) and a modifier (GCLM) subunit. The second important enzyme of GSH synthesis is GSH synthetase (GS) [21]. Our results revealed five differently expressed proteins related to GSH biosynthesis (GCLC, GSS, GPx1, GPx2, and GPx8) and two GSH transporters, ABCC4 (send GSH out of the cell) and SLC25A10 (send GSH in to the mitochondria). Many of GSH biosynthesis component significantly decreased, while the expression of glutamyl amino acid transporter (GGT7) increased. Probably cancer cells increase their transporter expression to compensate their GSH concentration from the culture media [22]. An increase in glutathione peroxidase 8 (GPx8) which act as H_2_O_2_ removal from the endoplasmic reticulum [23].

### Modulation of cell survival and stress response related signaling in the Nrf_2_^—^ cells

**Fig. 5:**
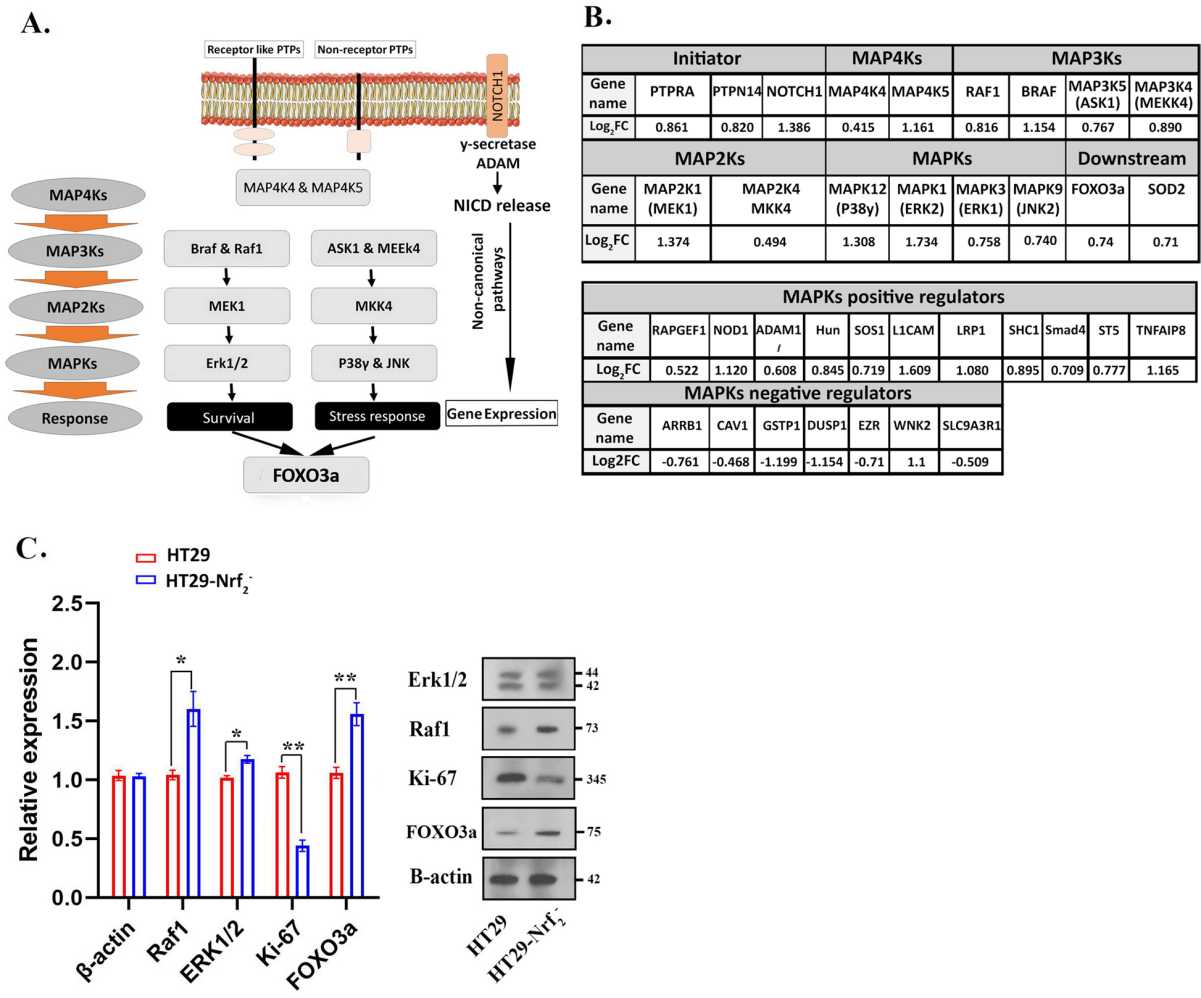
Over expressed MAPKs proteins followed by analyzing up regulated proteins in GO and KEGG pathway in HT29-Nrf_2_^−^ cells. **A** Proteins involved in activating MAPK signaling pathway and noncanonical Notch pathway. **B** ribosomal proteins and (**C**) transcriptional regulators as Log_2_ fold change (Log_2_FC) in Nrf_2_ knocked down cells. **D** Validation of MAPKs related proteins and downstream proteins (ERK1/2, Raf1 and FOXO3a) using Western blot analysis. As shown, MAPKs proteins were overexpressed while the expression of MAPKs negative regulators decreased. Results were expressed as the mean ± SD. (*p < 0.05, **p < 0.01, and ***p < 0.001)

The gene enrichment showed overexpression of different classes of MAPK proteins, which has been shown schematically in Fig. 5. Several receptors expressions increased such as NOTCH1 (2.6-fold), PTPRA receptor like (1.6-fold) and PTPN14 non-receptor protein tyrosine phosphatase (1.76-fold) which can initiate MAPK pathway (Fig. 5A, B). MAP4Ks (MAP4K4 and MAP4K5), MAP3Ks (Braf, Raf1, ASK1 and MEEK4), MAP2Ks or MAPKs are capable of initiating cellular survival by ERK1/ERK2 that have been increased in Nrf_2_ knock-downed cells. Moreover, the pathway is also regulated by several positive and negative regulators, which may affect the expression of MAPKs proteins (Fig. 5B). For data validation, we used western blot technique to quantify several final up and down stream proteins. Western blotting of RAF1 (MAP3Ks) which is an important initiator of MAPKs pathways revealed that this protein was up regulated by 1.7 ± 0.2 (*p*-value≤0.05) along with an increase in Erk1/2 expression (*p*-value≤0.05). Beside the activation of Erk1/2 survival pathway, stress response cascade proteins expression such as P38 and JNK were also increased. Furthermore, FOXO3a (a transcription factor) that plays an important role in the variety of cellular stress responses, increased by 1.6 ± 0.2 (*p*-value≤0.01) and previously was indicated within the most significantly enriched pathways in KEEG analysis. To distinguish between cell proliferation and survival, western blotting was done to examine the Ki-67 expression as a biomarker of tumor cell proliferation. Data showed a significant reduction of Ki-67 (*p* < 0.01) in HT29-Nrf_2_^−^ when compared with HT29 (Fig. 5C and Fig. 2S in supplementary data).

### Mitochondrial respiratory chain and RNA polymerase disabilities in the Nrf_2_^—^ cells

**Fig. 6:**
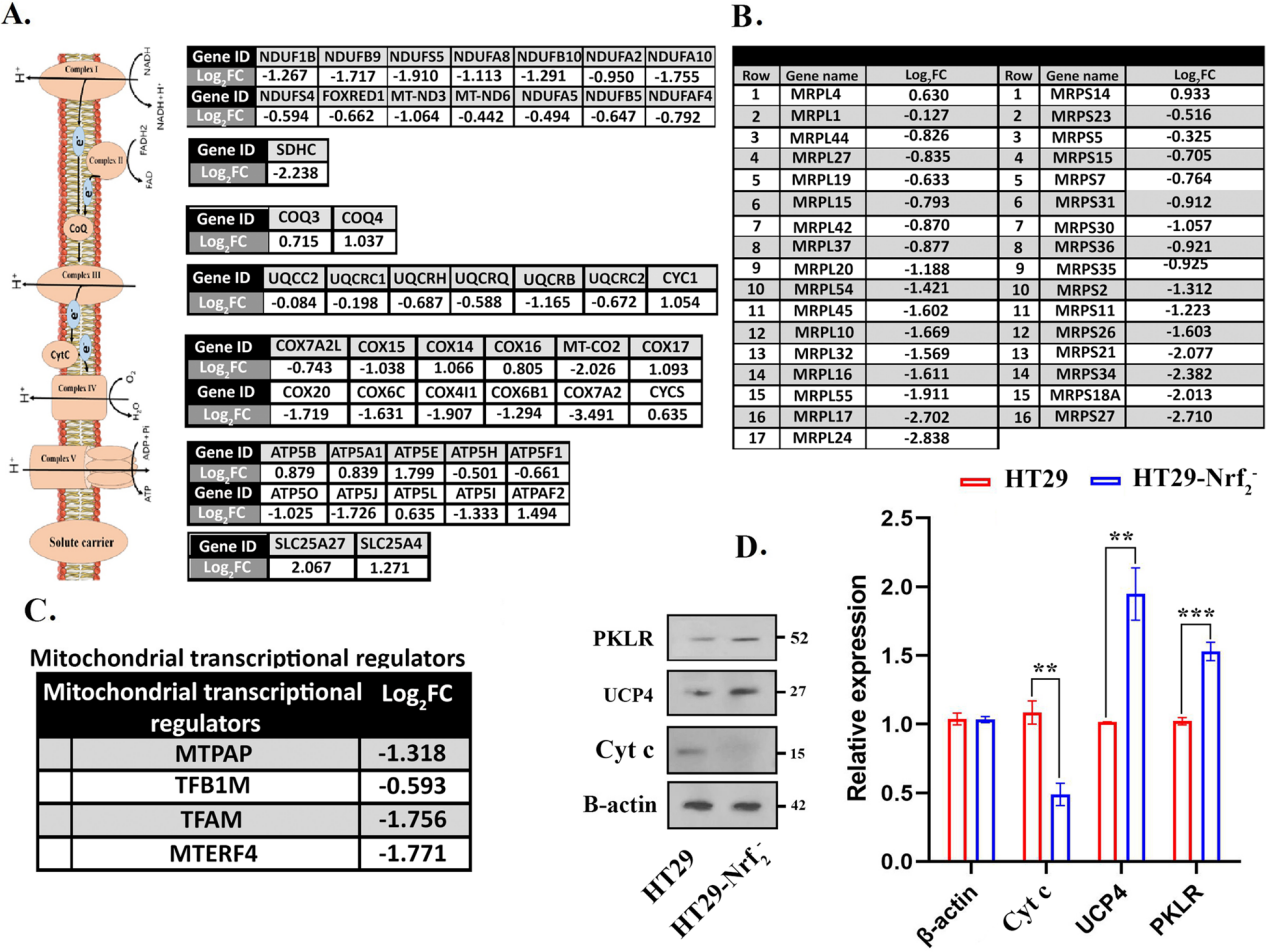
Investigating the expression of mitochondrial proteins in HT29-Nrf_2_^−^ cells. Down regulation of mitochondrial proteins related to (**A**) inner membrane and respiratory chain (**B**) large and small ribosomal subunits, presented as Log_2_Fold change (HT29-Nrf_2_^−^/HT29). **C** Decrease in mitochondrial transcriptional regulators in the absence of Nrf_2_ based on Log_2_FC. **D** Western blot analysis of PKLR, UCP-4 and Cyt c revealed that the expression of interest protein was significantly changed in HT29-Nrf_2_^−^ cells (**p < 0.01, and ***p < 0.001)

As mentioned above, mitochondrial ribosomes and respiratory chain were the most enriched terms in the gene ontology analysis. Forty-three proteins of mitochondria respiratory chain were extracted and listed from the proteomic data (Fig. 6A). In the mitochondria respiratory complex I, expression of NDUFs (NADH-ubiquinone oxidoreductase NADH: ubiquinone oxidoreductase subunit family’s) and other related proteins decreased more than 2-fold. The expression of succinate dehydrogenase (complex II) subunit (SDHC) also reduced remarkably. Ubiquinol Cytochrome c (Cyt c) Reductase (UQCR) proteins in complex III were also down regulated except cytochrome bc1 (CYC1) that was overexpressed and lead to a mitochondrial complex III dysfunction. In addition, proteins located in the final destination of electrons (Complex IV) were down regulated. Results revealed that proton transporter complex in mitochondria were collapsed in HT29-Nrf_2_^−^.

H+ potential produced by the respiratory complexes not only pass through the ATPase complex but it can also move across the inner membrane by some uncoupling proteins without producing ATP; consequently, it separates oxidative phosphorylation from ATP synthesis that significantly reduce ROS production. Here, an overexpression of uncoupling protein UCP4 (SLC25A27) was observed (Fig. 6D). The proteomic profile showed that six proteins in ATPase complex (ATP5H, ATP5F1, ATP5O, ATP5J, ATP5L and ATP5I) were down regulated and some of them (ATP5B, ATP5A1, ATP5E and ATPAF2) were up regulated. Surprisingly, over expression of UCP4 and ADP/ATP translocase (SLC25A4) along with the reduction of ATPase and respiratory chain complex was accompanied by an increase in the amount of two glycolysis enzymes, pyruvate kinase (PKLR) and hexokinase (HK2) with log_2_FC of 0.69 and 1.01 respectively. To confirm the proteomic data, western blotting was carried out on the both important glycolysis enzymes. Probably cells compensate their ATP shortage from the glycolysis pathway.

Main mitochondrial membrane proteins are expressed by 39 s/28 s mitoribosomes. The result showed that 16 large subunits of mitochondrial ribosomal (MRPLs) and 15 small subunits of mitochondrial ribosomal (MRPSs) were considerably down regulated (Fig. 6B). Interestingly the proteomic data demonstrated that the expression of mitochondrial transcriptional regulators including MTPAP, TFB1M, TFAM and MTERF4 were diminished in Nrf_2_ knocked down cells (Fig. 6C). In accordance with these data, Western blotting for Cyt c was done and the result showed a decrease in its expression in HT29-Nrf_2_^−^ cells when compared to HT29 cells (Fig. 6D and Fig. 3S in supplementary data).

### Protein-protein interaction analysis showed higher connection between nodes

**Fig. 7:**
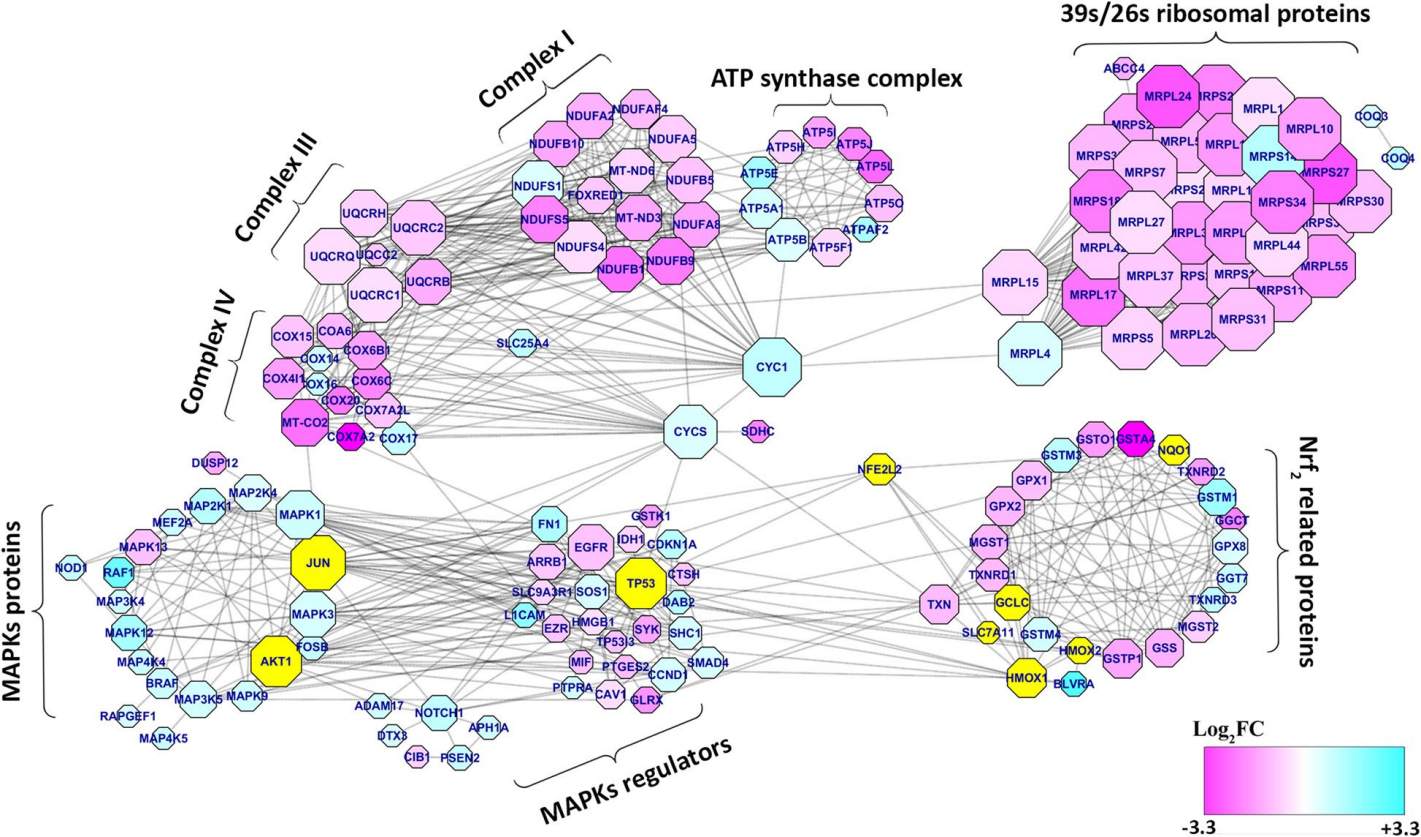
Coloration between Nrf_2_ related proteins and different cellular systems like MAPKs regulators and mitochondrial proteins were defined. The larger sizes of nodes are associated with more components and Log_2_FC expression that is differentiated by color from pink (− 3.3) as the lowest expression to blue (+ 3.3) as the highest expression ratio. The yellow color nodes are considered as proteins that have a direct relation with Nrf_2_

Based on primitive principal on big data analysis, individual proteins could not complete a series of actions in cellular physiology and thousands of proteins are required to make a complicated network to switch on/off a cellular response. Protein-protein interactions play a critical role in many essential cellular processes. Therefore, recognizing interactions are crucial for understanding the signaling events in cells under various conditions using well-known databases. These networks are typically represented by graphs with proteins as nodes and physical interactions by edges linking the nodes. Here, the PPI network was constructed using string database. In this network, 157 nodes (1 to 34 degree) were connected with edges and the crucial hub (a node with the highest degree) belonged to MRPL15 (39 s/26 s mitochondrial ribosomal subunit). The network exhibited that the 39 s/26 s mitochondrial ribosomal proteins have the most degree and CYC1, MRPL15, JUN, MT-CO2, TXN, MRPL4, TP53, COX15 and EGFR have the greatest betweenness. As presented in Fig. 7, eight important hub genes were identified and involved in previously mentioned pathways. In terms of biological process and molecular function, these hub genes are mainly enriched in mitochondrial electron transport chain, mitochondrial translational process and MAPKs pathways.

For better presentation of NFE2L2 position in the network, its first neighbors are illustrated as yellow color nodes and its betweenness located in the 27th place between 172 analyzed proteins (data not shown). These data confirmed that Nrf_2_ is not only a transcription factor of antioxidant defense, but also an important protein in colorectal cancer cell signaling and mitochondrial function.

## Discussion

Nowadays, an increment in ROS and oxidative stress in patients with advanced colorectal cancer, receiving chemotherapy and radiotherapy treatment, have been reported. In response to the oxidative stress, cancer cells enhance their antioxidant enzyme expression via activating various transcription factors like Nrf_2_ which is one of the most important cell antioxidant defenses.

Previous studies reported by Hong No and Wang noted the importance of Nrf_2_ in cellular drug resistance and showed that its activation could accelerate tumor metastasis [24, 25]. Due to the importance of Nrf_2_, its suppression and downstream pathways have been studied by many researchers, but the unknown signaling pathways which can help the cell to adopt ROS production to some extend were poorly recognized [26, 27]. Therefore, Nrf_2_ was knocked down in a colorectal cancer cell line, HT29, to study its effect on variety of events. Similar to other researches, results revealed a reduced viability and migration of HT29-Nrf_2_^−^ after subjected to two different types of chemotherapy drugs, doxorubicin and cisplatin (Fig. 1). Arlt and colleagues showed that inhibition of Nrf_2_ could increase the cancer cell susceptibility to drugs and promote apoptosis [28]. Many researchers clearly indicated doxorubicin as a DNA intercalator and ROS producer [29]. Although total ROS increased but the absence of Nrf_2_ only reduced the cell viability up to 20%, when compared to the control HT29 (Fig. 1). In the absence of drugs, reduction of Nrf_2_ did not affect the cell proliferation significantly and promoted the migration. Therefore, it was likely possible that Nrf_2_ knocked down cells were adopted to the ROS production. To explore the changes in signaling pathways, we decided to study HT29 and HT29-Nrf_2_^−^ proteome. For this reason, TMT labeling proteomic was carried out and differently expressed proteins were selected for functional annotation and cellular localization using KEGG and GO, respectively. Furthermore, PI network was constructed to screen hub genes involved in Nrf_2_ related gene network.

Several studies reported that the inhibition of Nrf_2_ could decrease the expression of proteins involved in oxidative stress or drug resistance like HO-1, NQO1, glutathione s-transferases, aldehyde dehydrogenases and thioredoxin related proteins [30]. Our proteomic data also revealed that the absence of Nrf_2_ in HT29-Nrf_2_^−^ is associated with reduction in GSH, HO-1, NQO1 and thioredoxin related antioxidant systems. A decrease in the antioxidant defense resulted in an increase in H_2_O_2_ that led to MDA production, a marker of lipid peroxidation. Several literatures have so far reported the pivotal role of Nrf_2_ in cell redox homeostasis [5, 31].

**Fig. 8:**
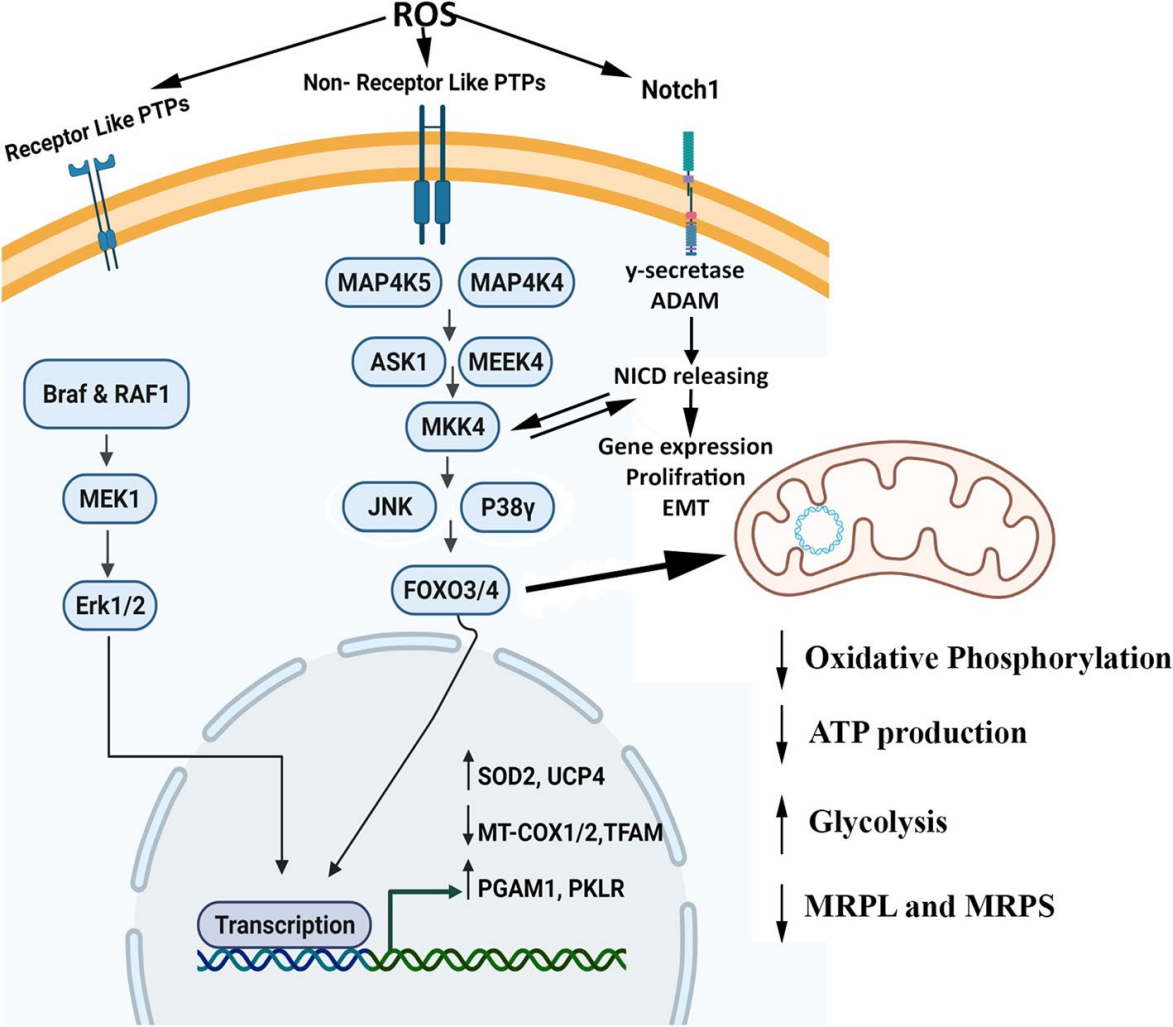
Overview of pathways elucidated by our research in HT29-Nrf_2_^−^ cells. In the absence of Nrf_2_, oxidative condition can activate several cell survival and stress response pathways. Based on our proteomic date, an increase in p38 and JNK and their upstream proteins could elevate the expression of FOXO3/4. FOXO protein translocation to the nucleus enhances SOD2 expression. In addition, FOXO protein can affect mitochondrial gene expression. Enhancement of Raf expression rise its downstream ERK 1/2 expression. Both ERK 1/2 and FOXO3/4 induces a specific set of genes involved in the regulation of various cellular processes. Also noncanonical Notch pathways may be activated by an increase in ROS and expression of 𝛾-secretase and ADAM 17 . Noncanonical Notch signaling interacts with MAPKs signaling and other pathways that could affect proliferation and EMT process

ROS appeared to be important second messengers that mediate different intracellular pathways with dual actions that regulate proliferation and apoptosis of cancer cells [32, 33]. Here, the wound healing assay exhibited the higher migration ability of HT29-Nrf_2_^−^ when compared to HT29 (*P* < 0.001). Previously researches reported that suppression of Nrf_2_ was associated with tumor cell plasticity and motility [34]. Therefore, we decided to elucidate the probable correlation between the downregulation of Nrf_2_ and migration. For this reason further analysis of the 171 differently expressed proteins were carried out. Up and down regulated genes were then subjected to KEGG and GO analysis (Figs. 2 and 3). The most up regulated proteins were associated to Notch, ErbB, FOXO and MAPKs pathways. Previously, a number of cellular stimuli that induced ROS production have also activated MAPK pathways in different cell types [35, 36]. MAPKs proteins were found to be involved in various biological process, molecular function and stress responses via three different signaling molecules: c-Jun N-terminal kinase (JNK), p38/MAPK, and ERK (Fig. 8) [37]. In the current study, an increase in p38/MAPK, JNK and ERK were observed in HT29-Nrf_2_^−^. It has been reported that MAPKs signaling pathway could activate survival cascade of urothelial cancer cells [38]. Both MEK and ERK1/2 have been involved in a wide range of processes, such as cell survival and proliferation [39]. Active Erk1/2 also plays a crucial role in the transit from G1 to S and cell cycle progression. Nowak et al. showed that under sub-lethal injury, Erk1/2 activation in renal proximal tubular cells can decrease basal respiration of mitochondria and ATP production which is a protective role that can inhibit prolonged injuries. In another way, survival following oxidant injury is associated with the activation of both ERK1/2 and JNK, whereas oxidant-induced death was associated with activation of JNK only [40].

In addition, several researches have showed that Notch transcription can be induced by Nrf_2_ (positive feedback), however, it can also be induced by non-canonical ways such as increased oxidative stress and activation of its cleavage [41–43]. Our proteomic results demonstrated an increase in Notch1 and enzymes (ADAM 17 (1.7-fold increase) and γ-secretase(1.7-fold increase)) related to its cleavage. Similar to our results, Notch pathway has been activated by MAPK signaling leading to proliferation and epithelial-mesenchymal transition (EMT) process, previously. Data from other studies also revealed that chemical inhibitors of secretase could inhibit the growth in colon cancer cell lines (HT29 and HCT116) via suppressing the Notch pathway [44–46]. However, some published results suggested that the effect of Notch signaling may dependent on the cell type or the treatment [46].

The increase in ROS also affected the expression of mitochondrial proteins. Within the mitochondria, ROS production declined the mitochondrial energy production by causing defects in mtDNA-encoded subunits of the respiratory complexes [47]. Mitochondrial DNA encodes 13 polypeptide of mitochondrial respiratory chain complexes, 22 tRNA and 2 rRNA (16 s and 12 s rRNA) [48]. Mitochondrial DNA transcription is controlled by nearly five nucleus encoded proteins: MTPAP (create the 3′ poly (A) tail for mitochondrial transcript), TFB1M (mitochondrial DNA promoter recognition), TFAM (mitochondrial transcriptional coactivator), MTERF4 (mitochondrial 39 s/28 s ribosome subunit assembly) and POLRMT (mitochondrial RNA polymerase) [49, 50]. Our proteomic data surprisingly showed that MTPAP, TFB1M, TFAM, MTERF4, 39 s/28 s mitochondrial ribosomal subunits and main proteins in respiratory chain (such as MT-ND3, MT-ND6 and MT-CO2) encoded by mitochondrial circular genome were down regulated (Fig. 6).

Since ROS is a by-product of normal mitochondrial respiration, respiratory chain proteins appeared to be reduced under oxidative stress to escape further cell damage. Elevated Hexokinase 2 and pyruvate kinase (Fig. 6D) in this study indicated that cells preferred glycolysis to mitochondria energetic function to deal with oxidative stress, which is in agreement with other data. Zhang et al. reported that loss of Nrf_2_ reduces mitochondria respiration and increases glycolysis to compensate ATP shortage [51].

In addition, the uncoupling protein 4 (UCP4) which is located in the inner membrane has increased probably due to its protective function against oxidative stress. Previously, researches have reported that the induction of mitochondrial uncoupling protein would lead to a decrease in ROS production [52]. Recent investigations have demonstrated that members of the UCP family can prevent mitochondrial ROS formation and oxidative stress. It has been reported that UCP transcriptional regulation occur via transcription factors with binding sites in the UCPs promoter [53], the peroxisome proliferator-activated receptor (PPAR) family [54], and forkhead transcription factors (FOXO). Its regulation has also been linked to fatty acid oxidation and oxidative stress [55]. Here, in the absence of Nrf_2_, an increase in FOXO3a, FOXO4, oxidative stress and glycolysis was observed that could be the reasons for the high expression of UPC4. Additionally, within differently expressed proteins, enhanced expression of SOD2 in HT29-Nrf_2_^−^ was seen. Researchers have reported that FOXO regulates detoxification of ROS via up regulation of mitochondrial superoxide dismutase (SOD2) [56]. FOXO pathway activation could also be the reason for a decrease in mitochondrial DNA copy number, expression of mitochondrial proteins, respiratory complexes and mitochondrial respiratory activity [57]. We assumed that stress induced expression of P38 MAPK and promoted FOXO3 nuclear localization that could be a key adaptive strategy (Fig. 8). Previous researchers reported that p38 regulates FOXO3a nuclear translocation and phosphorylates FOXO3a on Ser-7 upon doxorubicin treatment (as ROS producing drug) [58].

## Conclusions

Overall, absence of Nrf_2_ expression in HT29-Nrf_2_^−^ leads to a reduction of redox regulating proteins leading to an oxidative stress. Probably in the absence of ROS producing drugs, cells try to cope with this stress by increasing UCPs and SOD2 and affecting mitochondrial respiratory function and ROS production through MAPK and FOXO pathways. Since the production of mitochondrial ATP was reduced, glycolysis might have been used by cells for energy generation. Hence, signaling paths have dual role depending on the cell type, time and stimulus, thus, further investigation is required to determine the proteins with the most crucial role.

## Supplementary Information

**Additional file 1: Fig. 1S.** The original blot images of Fig. 1C in the manuscript. Western blot data of HO-1 (A) and β-actin (B) proteins. Left to right: marker, HT29 and HT29-Nrf2- cell lines. **Figure 2S.** The original blot images of Fig. 5C in the manuscript. Western blot data of Ki-67 (A), Erk1/2 (B), RAF1(C), FOXO3a (D) and β-actin (E) proteins. Left to right: marker, HT29 and HT29-Nrf2- cell lines. **Figure 3S.** The original blot images of Fig. 6D in the manuscript. Western blot data of PKLR (A), UCP4 (B), Cyt c (C), β-actin (D) proteins. In A, B and D; left to right: marker, HT29 and HT29-Nrf2- cell lines. In C; left to right: marker, HT29-Nrf2- and HT29 cell lines.

## Data Availability

Our Proteomic dataset with accession number “PXD027360” has been successfully submitted to ProteomeXchange via the PRIDE database. The reviewer can access to the data by this link https://www.ebi.ac.uk/pride/archive/projects/PXD027360. Also our proteomic raw data were uploaded in Related File section of journal submitting process which journal reviewer can consider this document.
