# Supplementary material for: The effect of Nrf2 deletion on the proteomic signature in a human colorectal cancer cell line
Source: BMC Cancer. 2022 Sep 13;22:979. doi: 10.1186/s12885-022-10055-y (PMC9472369; doi:10.1186/s12885-022-10055-y)
Supplement: Supplementary file 1 — Additional file 1: Fig. 1S. The original blot images of Fig. 1C in the manuscript. Western blot data of HO-1 (A) and β-actin (B) proteins. Left to right: marker, HT29 and HT29-Nrf2- cell lines. Figure 2S. The original blot images of Fig. 5C in the manuscript. Western blot data of Ki-67 (A), Erk1/2 (B), RAF1(C), FOXO3a (D) and β-actin (E) proteins. Left to right: marker, HT29 and HT29-Nrf2- cell lines. Figure 3S. The original blot images of Fig. 6D in the manuscript. Western blot data of PKLR (A), UCP4 (B), Cyt c (C), β-actin (D) proteins. In A, B and D; left to right: marker, HT29 and HT29-Nrf2- cell lines. In C; left to right: marker, HT29-Nrf2- and HT29 cell lines. [file 12885_2022_10055_MOESM1_ESM.docx]

**The effect of Nrf_2_ deletion on the proteomic signature in a human colorectal cancer cell line.**

Omid Cheraghi^1^. Bahareh Dabirmanesh^1*^. Farideh Ghazi^2^. Massoud Amanlou^3^.

Mona Atabakhshi-kashi^4^. Yaghoub Fathollahi^5^. Khosro Khajeh^1^

^1^ Department of Biochemistry, Faculty of Biological Science, Tarbiat Modares University,Tehran, Iran

^2^ Department of Medical Genetics and Molecular Biology, School of Medicine, Iran University of Medical Sciences, Tehran, Iran

^3^ Department of Medical Chemistry, Faculty of Pharmacy and Pharmaceutical Science, Tehran, Iran

^4^ CAS Key Laboratory for Biomedical Effects of Nanomaterials & Nanosafety, CAS Center for Excellence in Nanoscience, National Center for Nanoscience and Technology, Beijing 100190, China

^5^ Department of medical physiology, Faculty of Medical Science, Tarbiat Modares University,Tehran, Iran

***Correspondence to**:  Bahareh Dabirmanesh (Tarbiat Modares University, Tehran, Iran. P.O. Box: 14115-175. Tel: +98 21 82883428. Fax: +98 21 82884717. E-mail: [dabirmanesh@modares.ac.ir](mailto:dabirmanesh@modares.ac.ir))

**Original Western blot**

The original Western blots are presented below. Methods for these blots have been written in the manuscript.

.


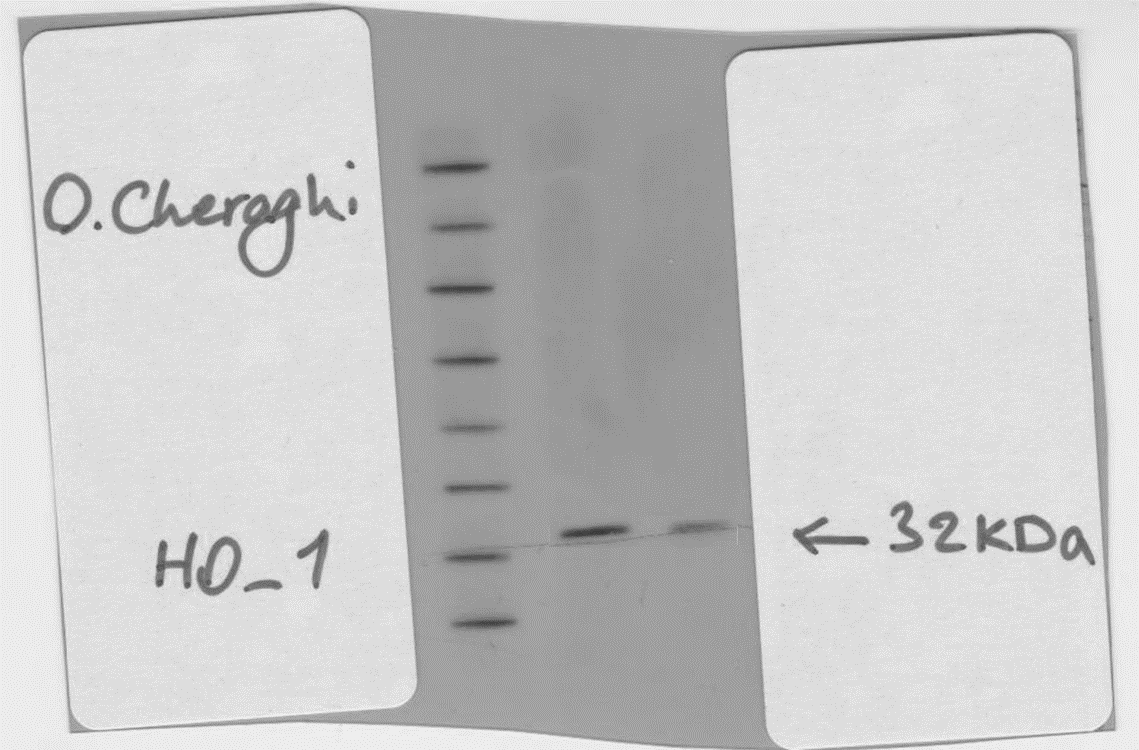


**A**


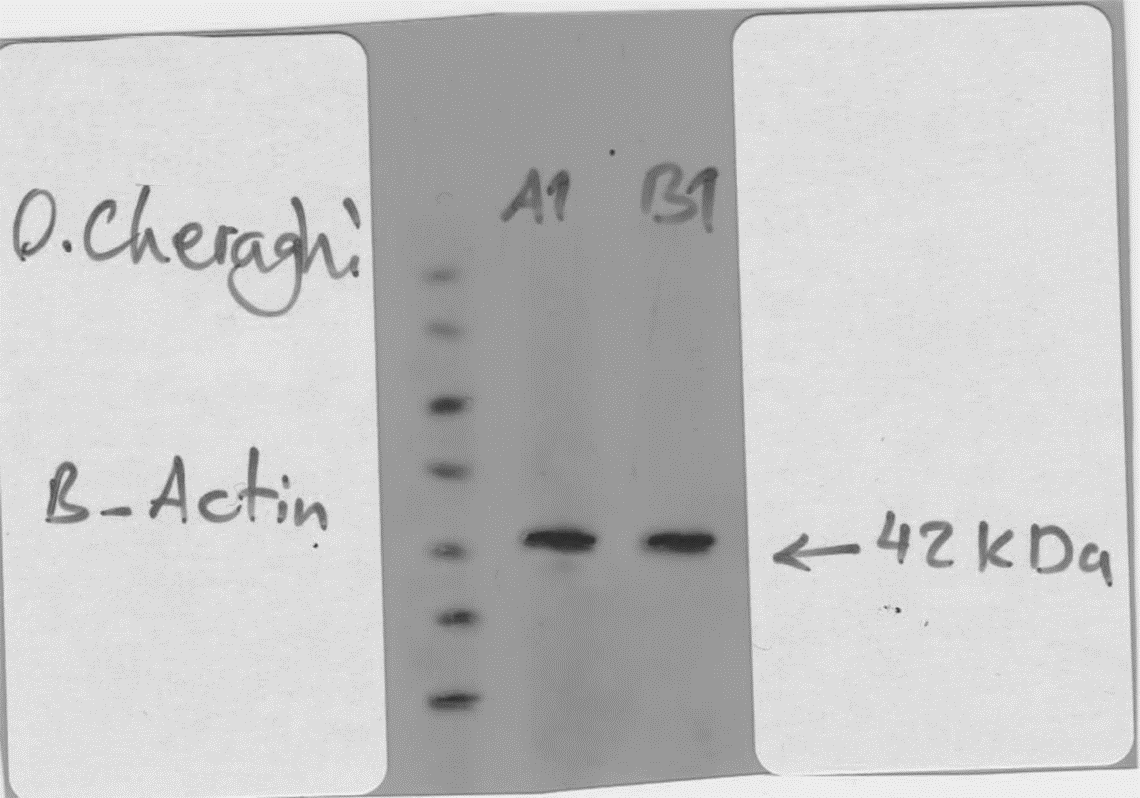


**B**

Fig. 1S) The original blot images of Fig. 1C in the manuscript. Western blot data of HO-1 (A) and β-actin (B) proteins. Left to right: marker, HT29 and HT29-Nrf_2_^-^ cell lines.


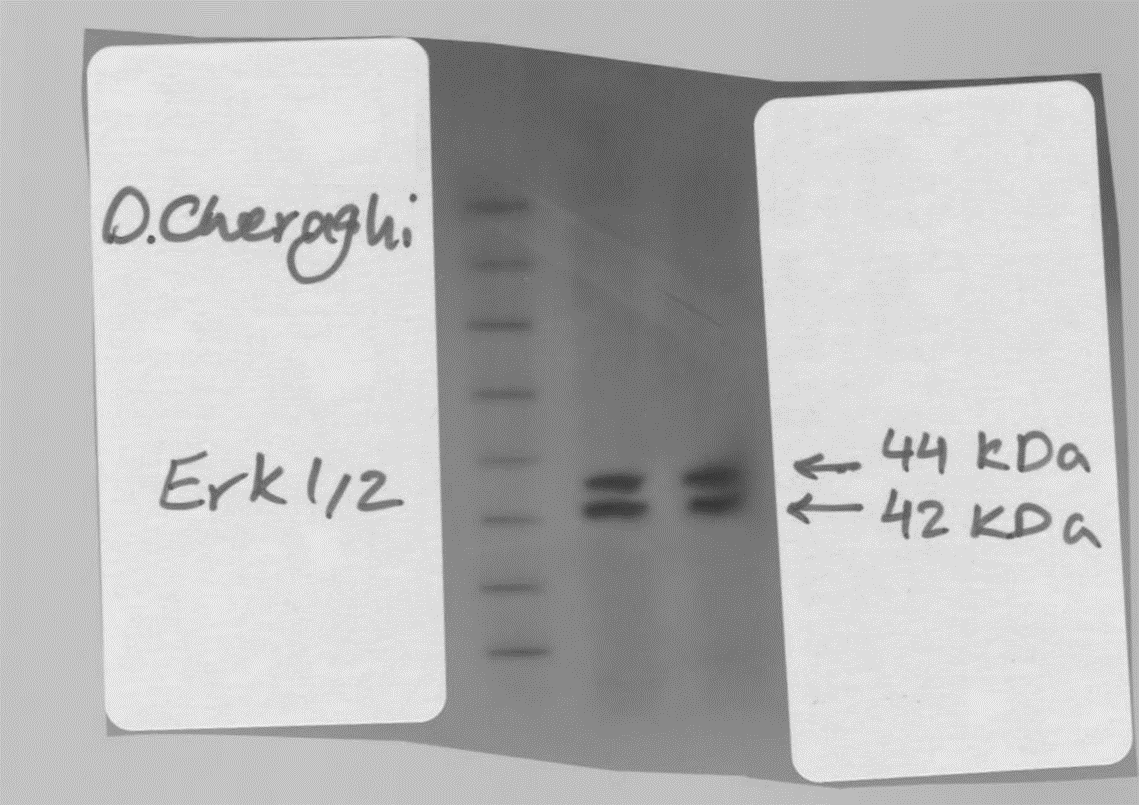

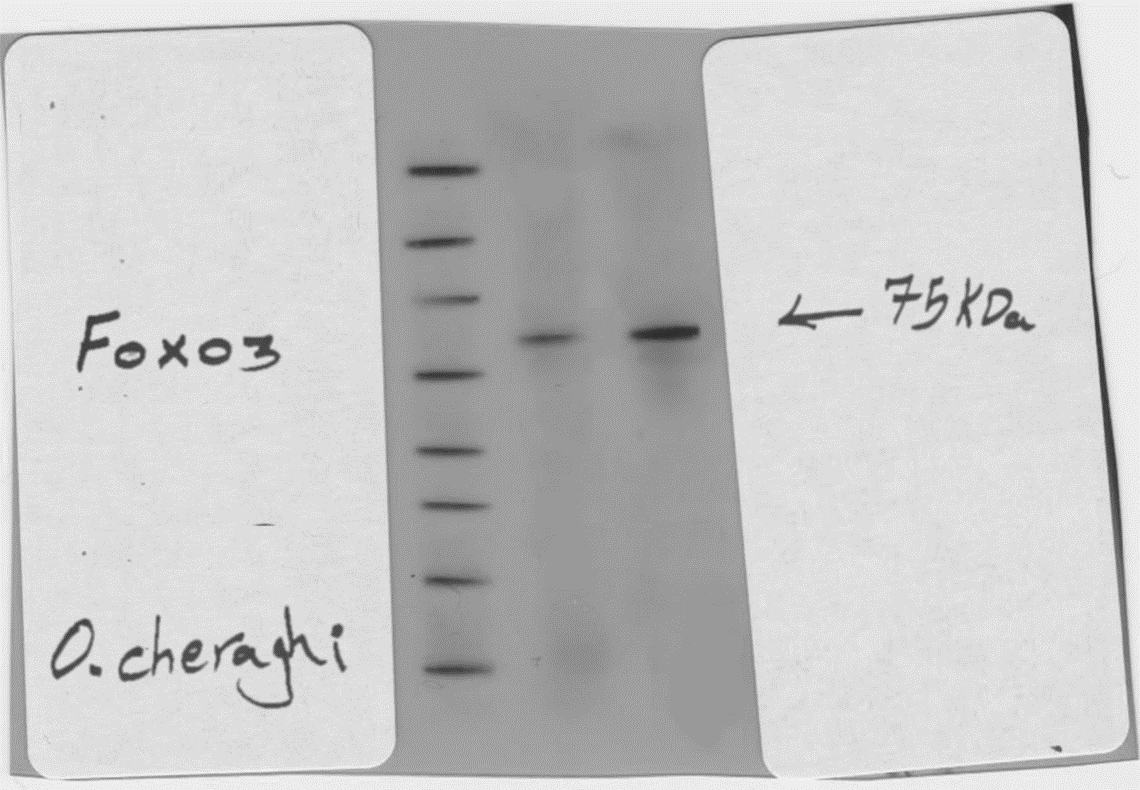

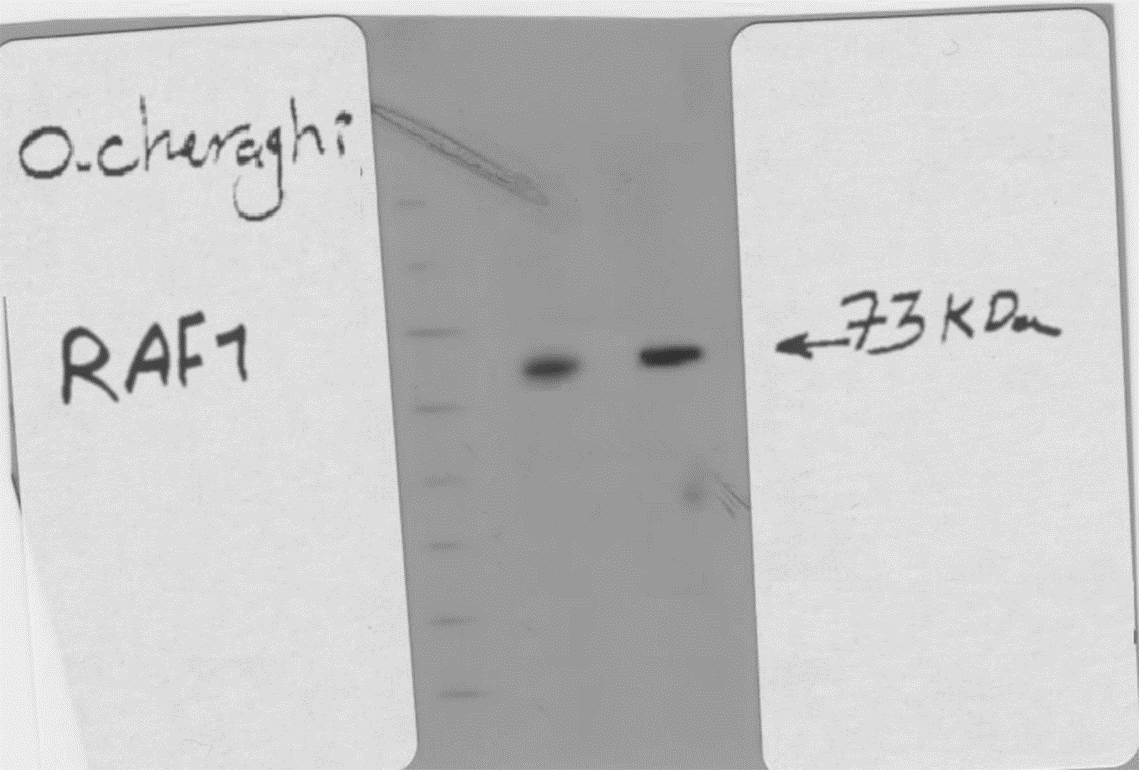

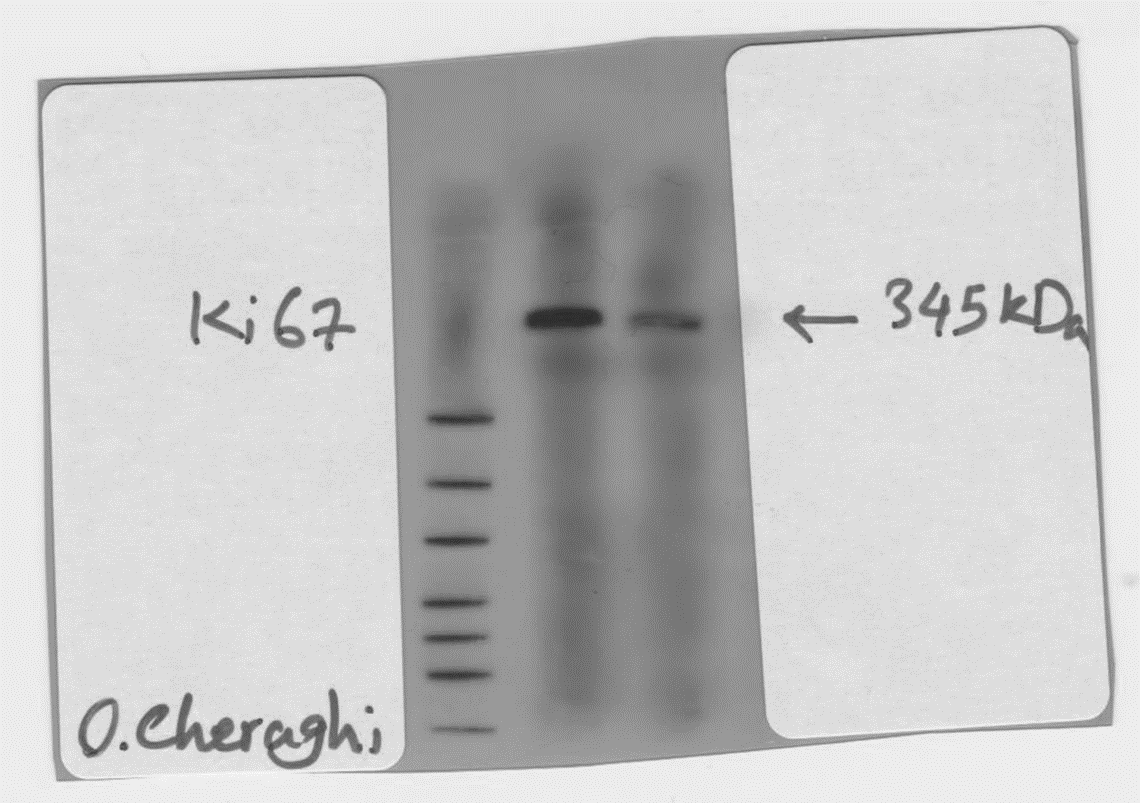


**C**

**D**

**B**

**A**


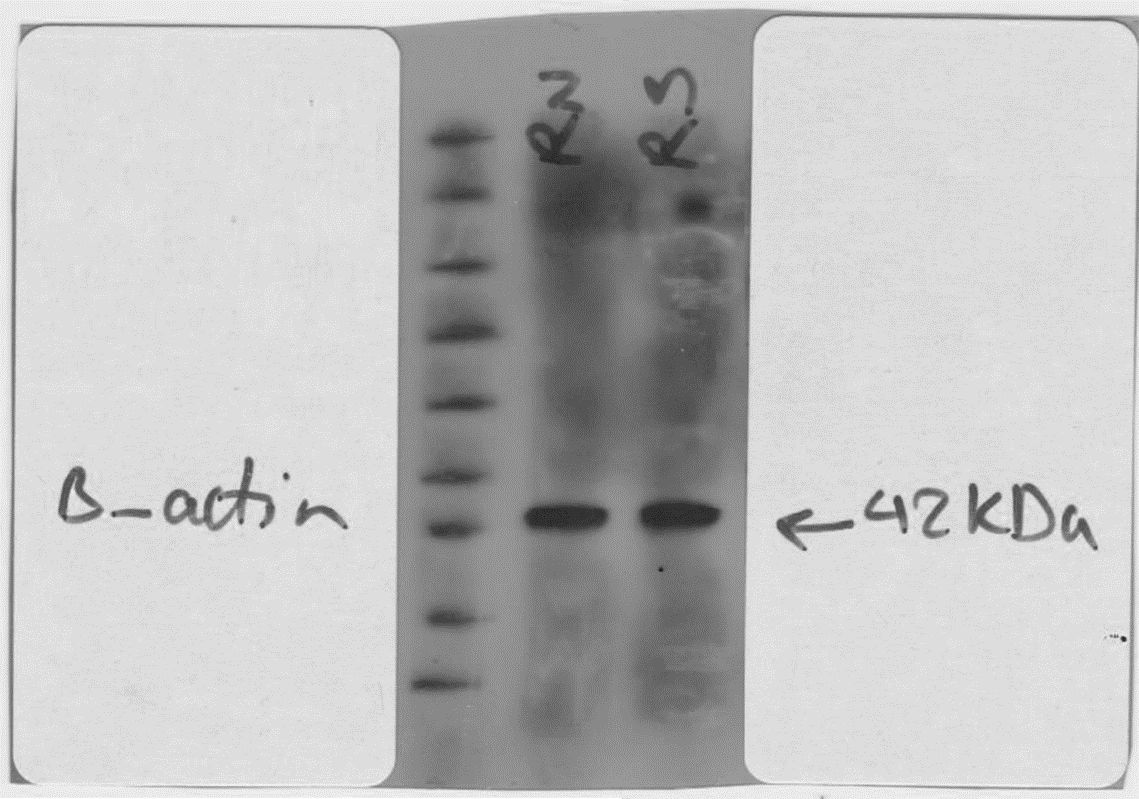


**E**

1-HT29

2-HT29-Nrf2-

Fig. 2S) The original blot images of Fig. 5C in the manuscript. Western blot data of Ki-67 (A), Erk1/2 (B), RAF1(C), FOXO3a (D) and β-actin (E) proteins. Left to right: marker, HT29 and HT29-Nrf_2_^-^ cell lines.


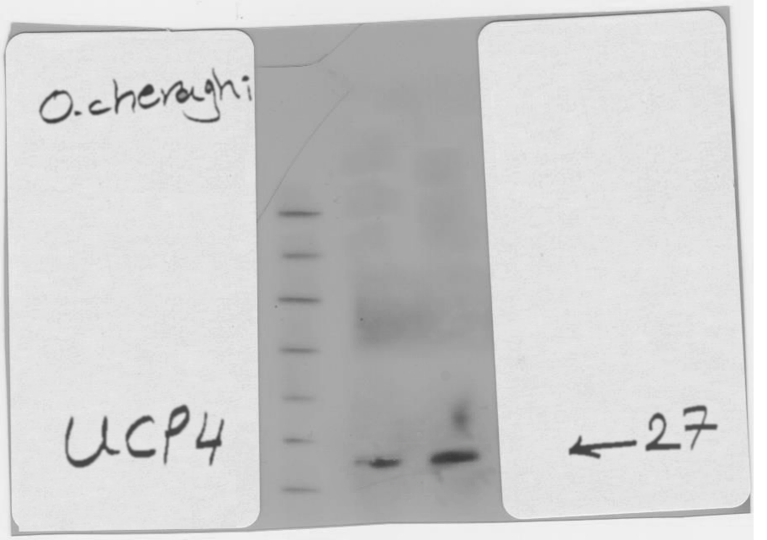

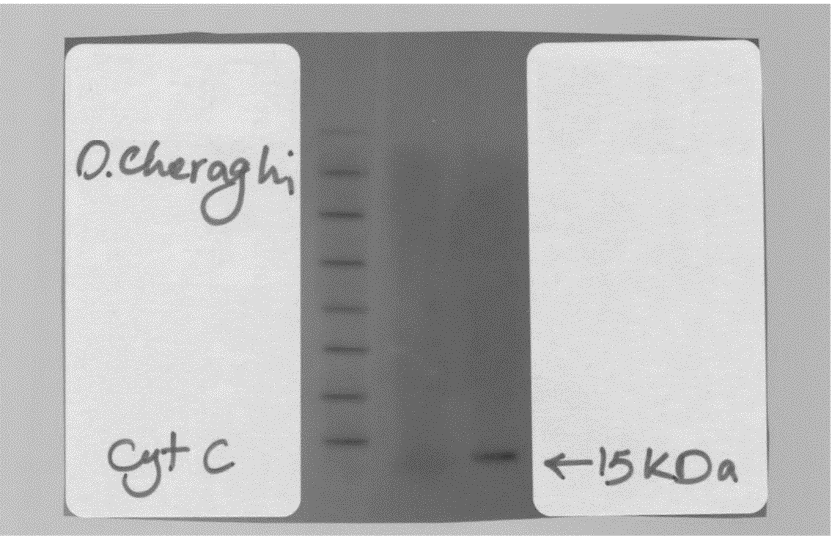

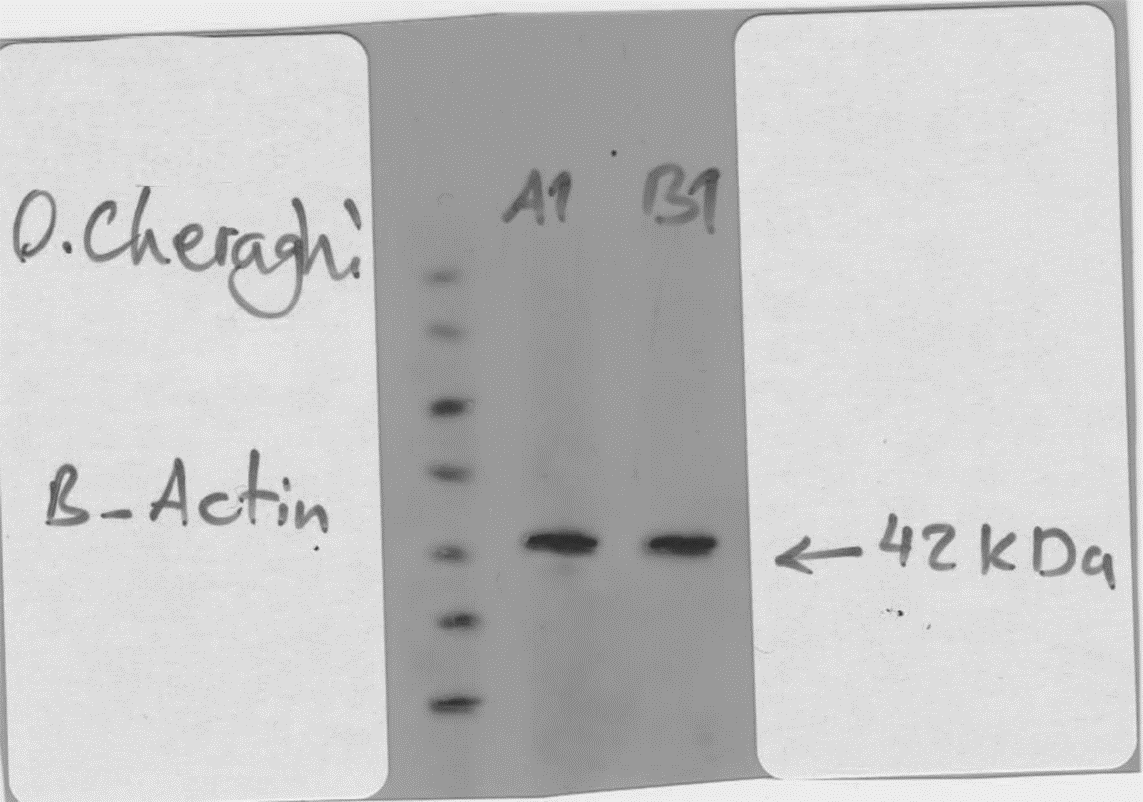

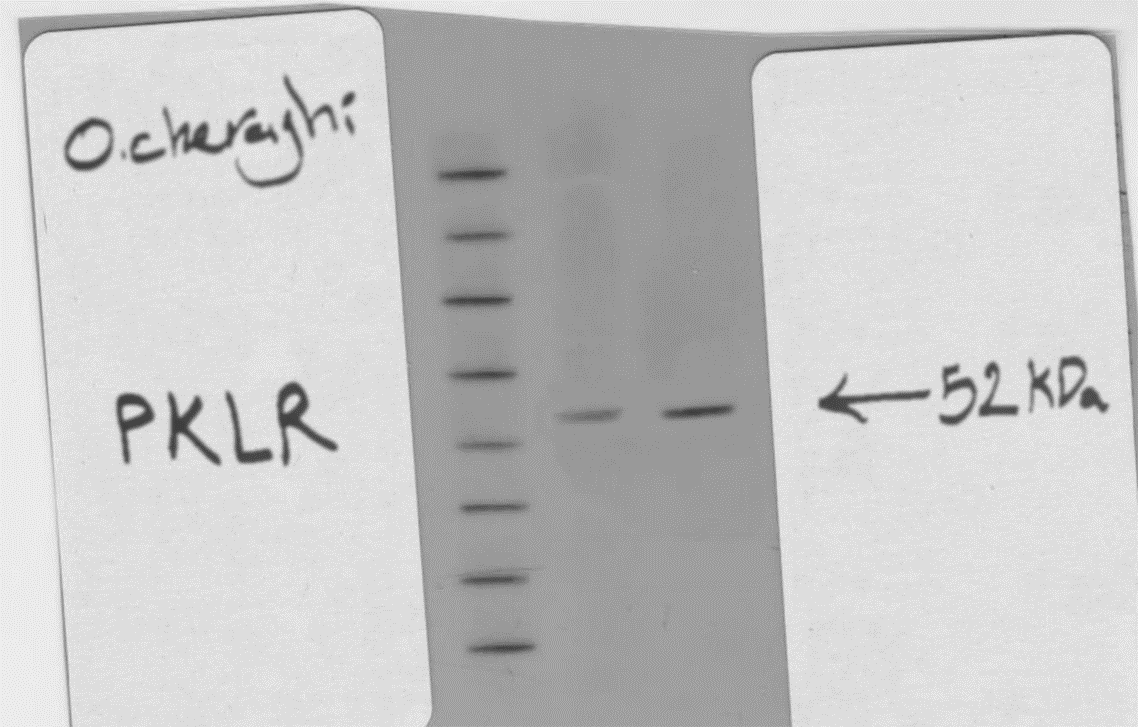


**B**

**A**

**C**

**D**

**B**

**A**

Fig. 3S) The original blot images of Fig. 6D in the manuscript. Western blot data of PKLR (A), UCP4 (B), Cyt c (C), β-actin (D) proteins. In A, B and D; left to right: marker, HT29 and HT29-Nrf_2_^-^ cell lines. In C; left to right: marker, HT29-Nrf_2_^-^ and HT29 cell lines.
